# Supplementary material for: Effect of robotic-assisted gait training on gait and motor function in spinal cord injury: a protocol of a systematic review with meta-analysis
Source: BMJ Open. 2023 Sep 22;13(9):e070675. doi: 10.1136/bmjopen-2022-070675 (PMC10533792; doi:10.1136/bmjopen-2022-070675)
Supplement: Author's manuscript [file bmjopen-2022-070675.draft_revisions.pdf]

BMJ Open is committed to open peer review. As part of this commitment we make the peer review history of every article we publish publicly available.

When an article is published we post the peer reviewers' comments and the authors' responses online. We also post the versions of the paper that were used during peer review. These are the versions that the peer review comments apply to.

The versions of the paper that follow are the versions that were submitted during the peer review process. They are not the versions of record or the final published versions. They should not be cited or distributed as the published version of this manuscript.

BMJ Open is an open access journal and the full, final, typeset and author-corrected version of record of the manuscript is available on our site with no access controls, subscription charges or pay-per-view fees (http://bmjopen.bmj.com).

If you have any questions on BMJ Open's open peer review process please email info.bmjopen@bmj.com

BMJ Open

Effect of robotic-assisted gait training on gait and motor function in spinal cord injury: a protocol of a systematic review with meta-analysis

Journal:	*BMJ Open*
Manuscript ID	bmjopen-2022-070675
Article Type:	Protocol
Date Submitted by the Author:	05-Dec-2022

Complete List of Authors:	wang, lei; Hunan Provincial People's Hospital,<br>peng, lin; Tongji Hospital of Tongji Medical College of Huazhong University of Science and Technology<br>chen, lian; Hunan Provincial People's Hospital

Keywords:	REHABILITATION MEDICINE, NEUROSURGERY, Neurological injury < NEUROLOGY

SCHOLARONE™
Manuscripts

Effect of robotic-assisted gait training on gait and motor function in spinal cord injury: a protocol of a systematic review with meta-analysis

Name	Affiliation	Email
Lei Wang	Department of Rehabilitation Medicine, Hunan Provincial People's Hospital, The First Affiliated Hospital of Hunan Normal University, Changsha 410005, Hunan Province, China.	wangleipx168@163.com
Jin-lin Peng	Tongji Hospital, Tongji Medical College, Huazhong University of Science & Technology, Wuhan 430000, Hubei Province, China.	pjlkim@163.com
Ai-lian Chen	Department of Rehabilitation Medicine, Hunan Provincial People's Hospital, The First Affiliated Hospital of Hunan Normal University	652326303@qq.com

First author:

Lei Wang, wangleipx168@163.com, Department of Rehabilitation Medicine, Hunan Provincial People's Hospital (The First Affiliated Hospital of Hunan Normal University), Changsha 410005, Hunan, Peoples R China

Corresponding Author:

Ai-lian Chen, 652326303@qq.com , Department of Rehabilitation Medicine, Hunan Provincial People's Hospital (The First Affiliated Hospital of Hunan Normal University), Changsha 410005, Hunan, Peoples R China.

For peer review only

Effect of robotic-assisted gait training on gait and motor function in spinal cord injury: a protocol of a systematic review with meta-analysis

Abstract：

Introduction: Robotic-assisted gait training (RAGT) has been reported to be effective in the rehabilitation of patients with spinal cord injury (SCI). However, studies on RAGT showed different results because they varied in terms of the number of samples. Thus, summarizing studies based on robotic-related factors is critical for the accurate estimation of the effects of RAGT on SCI. This work aims to search for strong evidence showing that using RAGT is effective in the treatment of SCI and to analyze the deficiencies of current studies.

Methods and analysis: The following publication databases were electronically searched in December 2022 without restrictions on publication year: Medline, Cochrane Library, Web of Science, Embase, PubMed, and China National Knowledge Infrastructure. All articles on randomized controlled trials using RAGT to treat SCI that were published in English and Chinese and met the following criteria will be included. Outcomes included motor function, and gait parameters included those assessed by using instrumented gait assessment, the Berg balance scale, the 10 m walking speed test, the 6 min walking endurance test, the functional ambulation category scale, the Walking index of SCI, and the ASIA assessment scale. Research selection, data extraction, and quality assessment will be conducted independently by two reviewers to ensure that all relevant studies are free from personal bias. The Cochrane Bias Risk Assessment Tool will be used to assess the risk of bias. Review Manager V.5.3 software will be utilized to produce deviation risk maps and perform paired meta-analyses.

Strengths and limitations of this study

For peer review only

1.This study will be the first meta-analysis to systematically evaluate the efficacy and safety of RAGT in the treatment of SCI.

2.The results of this study will provide evidence for the treatment of SCI patients, and help therapists and patients to choose appropriate treatment methods.

3.To ensure that all relevant studies are free from personal bias, two reviewers will independently conduct research selection, data extraction and quality assessment.

4.The language categories of the research search are only included in English and Chinese, and the final search results will have some bias.

Key words: Spinal Cord Injuries; Motor disorders; Rehabilitation; Robotics, Gait Analysis

Ethics and dissemination: Ethics approval is not required for systematic reviews and network meta-analyses. The results will be submitted to a peer-reviewed journal or presented at a conference.

Trial registration number: PROSPERO (CRD42022319555).

Introduction

Spinal cord injury (SCI) is a serious disabling disease that often causes paraplegia or quadriplegia and affects the patient's sensory, motor, and autonomic nervous functions[1]. SCI leads to a variety of complications, such as pressure ulcers, lung infections, and urinary tract infections[2]. Moreover, it affects the quality of life and living standard of patients and imposes a heavy burden on families[3] and society. It ultimately shortens the life expectancy of patients[4]. National statistical data show that the incidence rate of SCI is increasing annually and that the incidence rate of TSCI per million residents is 9.3 persons/year[5]. During the rehabilitation treatment of SCI, improving the walking ability, self-care ability, and self-esteem of patients is an important aspect that helps patients return to society and reduces their costs. Therefore, the rehabilitation of the lower limbs, which mainly

For peer review only

function in standing and walking, is crucial.

Robot-assisted gait training (RAGT) can improve the walking ability[6], lower limb strength, and independence of patients with incomplete SCI[7]. It can also improve balance function[8]. RAGT has been gradually applied in patients with SCI. Some clinical evidence shows that in patients with SCI, robots for lower limb rehabilitation can effectively and safely improve walking ability; reduce pressure ulcers, lung infections, urinary tract infections, and other complications; improve dignity; and reduce costs. However, high-quality evidence-based medical studies that systematically evaluated the efficacy of RAGT in the treatment of SCI remain scarce.

Summarizing studies based on RAGT-related factors is critical for the accurate estimation of the effects of RAGT on SCI. This meta-analysis aims to evaluate systematically the efficacy of RAGT in alleviating motor dysfunction and restoring speech ability in patients with SCI according to randomized clinical trials (RCTs); find strong evidence demonstrating that using RAGT is effective in the treatment of SCI; and analyze the deficiencies of current studies.

Methods

The protocol of this systematic review was planned and conducted in accordance with the Preferred Reporting Items for Systematic Reviews and Meta-Analyses Protocols Guideline and Cochrane Collaboration[9]. The review process is shown in Figure

Search strategy

Two reviewers (Jin-lin Peng and Lei Wang) electronically searched the following publication databases in December 2022 without restrictions on publication year: Medline, Cochrane Library, Web of Science, Embase, PubMed, and China National Knowledge Infrastructure. Various combinations of keywords, including “motor disorders,” “robotics”, “robotic assisted gait training,”

For peer review only

“noninvasive brain stimulation,” “SCI”, and “gait analysis”, were used as search terms. The key terms matched the appropriate Medical Subject Headings terms. Presearches were performed. Then, the final search was conducted follows: Relevant journals and references of review articles were manually searched online to identify papers that may have been missed in the electronic database searches.

Eligibility criteria

Inclusion criteria

(1) Study design: Only RCTs were included. (2) Selected population: Participants diagnosed with SCI, namely, individuals with any level of traumatic SCI, regardless of the time since injury, sex, and age, were included. (3) Type of intervention: The experimental groups received tDCS or tDCS combined with other physical therapies. The control group received sham tDCS or other types of physical therapy. (4) Comparison: The treated subjects were compared at baseline then with the control or sham-stimulated subjects. (5) Type of outcomes measured: Gait analysis indicators, including gait speed (m/s), step length (cm), double support phase (% walking cycle), single support phase (% walking cycle), and symmetry index; Berg balance scale; ASIA assessment scale; Holden walking ability classification (functional ambulation category scale); 10 m walking speed test; 6 min walking endurance test; and WISCI Ⅱ score.

Exclusion criteria

Studies involving animal research, conference research, protocol studies, or computer model research and duplicate papers were excluded. Two reviewers (Jin-lin Peng and Lei Wang) independently screened titles and abstracts to identify articles reporting studies that met the inclusion criteria. Then, the full-text versions of the identified articles were obtained and separately

For peer review only

screened to ensure that they met the inclusion criteria. A third reviewer (Ai-lian Chen) made the final assessment regarding whether or not full-text papers met the inclusion criteria.

Data extraction

A reviewer (Lei Wang) prepared the general information and data collection process by another reviewer (Jin-lin Peng). The format of data collection included the following factors: research design, participants (number, diagnosis, age, and target population numbers in each group), eligibility criteria, intervention used on the research group and control group (i.e., site of stimulation, intensity, number of sessions, and time of each session), and outcomes of interest.

Quality assessment

The quality evaluation of the included studies was performed independently by two reviewers (Jin-lin Peng and Lei Wang) and was revised by the third reviewer (Ai-lian Chen). The methodological quality of the intervention studies was assessed by using the Physiotherapy Evidence Database (PEDro) scale. The PEDro scale is a valid and reliable measure of the methodological quality of RCTs. This 10-item scale is based on the core criteria for RCT quality assessment[10]. The quality of papers was classified as follows in accordance with the PEDro scale: Studies with scores of less than 6 points were considered low-quality studies, whereas those with scores equal to or greater than 6 points were considered high-quality studies (where scores of 6–7 indicate good quality and those of 8–10 indicate excellent quality)[11].

The GRADEpro GDT online tool was used to evaluate the level of evidence quality of the outcome indicators. The tool is available at its official website http://www.guidelinedevelopment.org/. The GRADEpro GDT online tool for evaluating the quality of outcome indicators includes five degrading factors: risk of bias, inconsistency, indirectness, imprecision, and other considerations.[12]

For peer review only

The quality of evidence can be divided into four levels of "high", "moderate", "low", and "very low."[13]

Risk-of-bias assessment of individual studies

The quality of the included studies was evaluated and their scores were compared in a consensus meeting between two independent authors (Jin-lin Peng and Lei Wang) to minimize errors and potential biases in the evaluation. In the event of any disagreement, a third author (Ai-lian Chen) was included in the discussion for a final consensus. The Cochrane risk-of-bias assessment tool outlined in Chapter 8 of the *Cochrane Hand-book for Systematic Reviews of Interventions* (Version 5.1.0) was used to assess the risk of bias of the articles. Each article was assessed for selection bias (random sequence generation and allocation concealment), performance bias (blinding of participants and personnel), detection bias (blinding of outcome assessment), attrition bias (incomplete outcome data reporting), and reporting bias (selective outcome reporting). Each domain was rated as high risk of bias, unclear of bias, or low risk of bias. The risk map of the biases of the studies' quality was prepared with RevMan 5.2 software.

Patient and public Involvement

No patient participated in writing the system review plan. However, the results will be disseminated to patients with SCI.

Statistical analysis

A meta-analysis will be conducted by using Review Manager 5.3. Heterogeneity between studies will be evaluated on the basis of the I^2 statistic for the quantification of the proportion of the total outcome attributable to variability among studies. The following ranges were defined: $I^2 = 0\%–30\%$ (no heterogeneity), $I^2 = 30\%–49\%$ (moderate heterogeneity), $I^2 = 50\%–74\%$ (substantial

For peer review only

heterogeneity), and I^2 = 75%–100% (considerable heterogeneity)[14]. On the basis of heterogeneity, a random-effects model was used when $I^2 > 30\%$, and a fixed-effects model was utilized when I^2 = 0%–30%.

For the comparison of data from different scales, pooled statistics will be calculated by using standardized mean differences (SMDs). Means and standard deviations after intervention and follow-up evaluation for the RAGT and control groups (when relevant) will be applied to compute SMDs.

Addressing missing data

The original author will be contacted for additional information regarding missing data. In the absence of a reply, the data will be calculated on the basis of the availability factor. The potential effect of the missing data on meta-analysis results will be tested through sensitivity analysis.

Subgroup analysis

Grouping analysis will be performed to address potential heterogeneity and inconsistencies and will be conducted in accordance with age, gender, SCI plane, disease course, treatment prescription, and treatment duration. At the same time, meta-analysis will be conducted to explore the possible sources of heterogeneity.

Sensitivity analysis

For the verification of the robustness of the research conclusion, sensitivity analysis will be conducted on the main results to assess the effect of method quality, research quality, sample size, missing data, and analysis methods on the results of this review[15].

Assessment of publication bias

Each included study will be evaluated in accordance with the PEDro scale. Funnel charts will be

For peer review only

used to assess the publication bias of the main results included in the study. If the funnel chart is found to be asymmetrical, attempts will be made to explain its asymmetry [16].

Discussion

RAGT can improve the walking ability of patients with incomplete SCI and can be used by patients with stable vital signs. For patients with complete SCI, RAGT acts mainly to maintain the range of motion of joints. In recent years, studies on using RAGT to improve walking ability in SCI have increased, and the new exoskeleton robot for lower limb rehabilitation has shown the advantage of safe transfer. Our current query shows that our work is the first systematic review and meta-analysis on RAGT for patients with SCI. The results of this meta-analysis can help patients and therapists select the appropriate treatment method for SCI and improve new options on the basis of the comparative evidence for effectiveness and safety. We hope that the results of this study will provide evidence for guideline recommendations.

Data Availability

The datasets used and analyzed in the current study are included in this article.

Ethical Approval

This research is a review, does not involve ethical issues, and did not apply for ethical approval.

Funding

This study has no funding support.

Disclosure

All authors have read and approved the final manuscript.

Contributors

WL and P-JL, as the first authors, have made equal contributions to this work. Research concept and design: WL and C-AL. Data acquisition: WL and P-JL. Draft: WL and P-JL. Supervised by: C-AL. All the authors approved the publication of the Protocol.

For peer review only

Conflicts of Interest

All authors declare no potential conflicts of interest with respect to the research, authorship, and/or publication of this study.

Reference

[1] Eckert MJ, Martin MJ. Trauma: Spinal Cord Injury[J]. Surg Clin North Am, 2017,97(5):1031-1045. DOI: 10.1016/j.suc.2017.06.008.

[2] Stricsek G, Ghobrial G, Wilson J, et al. Complications in the Management of Patients with Spine Trauma[J]. Neurosurg Clin N Am, 2017,28(1):147-155. DOI: 10.1016/j.nec.2016.08.007.

[3] Zhang JM, Li N, Zhu L, et al. Effects of pelvic floor biofeedback electrical stimulation combined with lower limb rehabilitation robot training on intestinal function of patients with spinal cord injury [J]. Journal of Brain and Nervous Diseases,2021,29(01):53-57.

[4] Xiang XN, Zhong HY, He HC. Research progress of lower limb exoskeleton rehabilitation robot in improving walking ability of patients with spinal cord injury [J]. Chinese Journal of Rehabilitation Medicine,2020,35(01):119-122. DOI：CNKI:SUN:ZGKF.0.2020-01-024

[5] Bárbara-Bataller E, Méndez-Suárez JL, Alemán-Sánchez C, et al. Change in the profile of traumatic spinal cord injury over 15 years in Spain[J]. Scand J Trauma Resusc Emerg Med, 2018,26(1):27. DOI: 10.1186/s13049-018-0491-4.

[6] Grasmücke D, Zieriacks A, Jansen O, et al. Against the odds: what to expect in rehabilitation of chronic spinal cord injury with a neurologically controlled Hybrid Assistive Limb exoskeleton. A subgroup analysis of 55 patients according to age and lesion level[J]. Neurosurg Focus, 2017,42(5):E15. DOI: 10.3171/2017.2.FOCUS171.

[7] Holanda LJ, Silva P, Amorim TC, et al. Robotic assisted gait as a tool for rehabilitation of individuals with spinal cord injury: a systematic review[J]. J Neuroeng Rehabil, 2017,14(1):126. DOI: 10.1186/s12984-017-0338-7.

[8] Nam KY, Kim HJ, Kwon BS, et al. Robot-assisted gait training (Lokomat) improves walking function and activity in people with spinal cord injury: a systematic review[J]. J Neuroeng Rehabil, 2017,14(1):24. DOI: 10.1186/s12984-017-0232-3.

[9] Cumpston M, Li T, Page MJ, et al. Updated guidance for trusted systematic reviews: a new edition of the Cochrane Handbook for Systematic Reviews of Interventions[J]. Cochrane Database Syst Rev, 2019,10:ED000142. DOI: 10.1002/14651858.ED000142.

[10] Elbanna ST, Elshennawy S, Ayad MN. Noninvasive Brain Stimulation for Rehabilitation of Pediatric Motor Disorders Following Brain Injury: Systematic Review of Randomized Controlled Trials[J]. Arch Phys Med Rehabil, 2019,100(10):1945-1963. DOI: 10.1016/j.apmr.2019.04.009.

[11] Maher CG, Sherrington C, Herbert RD, et al. Reliability of the PEDro scale for rating quality of randomized controlled trials[J]. Phys Ther, 2003,83(8):713-721.

[12] Nasser M, Fedorowicz Z. Grading the quality of evidence and strength of recommendations: the GRADE approach to improving dental clinical guidelines[J]. J Appl Oral Sci, 2011,19(1):0. DOI: 10.1590/s1678-77572011000100001.

[13] Brożek JL, Akl EA, Compalati E, et al. Grading quality of evidence and strength of recommendations in

clinical practice guidelines part 3 of 3. The GRADE approach to developing recommendations[J]. Allergy, 2011,66(5):588-595. DOI: 10.1111/j.1398-9995.2010.02530.x.

[14] Higgins JP, Thompson SG. Quantifying heterogeneity in a meta-analysis[J]. Stat Med, 2002,21(11):1539-1558. DOI: 10.1002/sim.1186.

[15] Li J, Zhong D, Ye J, et al. Rehabilitation for balance impairment in patients after stroke: a protocol of a systematic review and network meta-analysis[J]. BMJ Open, 2019,9(7):e026844. DOI: 10.1136/bmjopen-2018-026844.

[16] Sterne JA, Sutton AJ, Ioannidis JP, et al. Recommendations for examining and interpreting funnel plot asymmetry in meta-analyses of randomised controlled trials[J]. BMJ, 2011,343:d4002. DOI: 10.1136/bmj.d4002.

Figure: Flow chart of meta-analysis for robotic-assisted gait training in patients with spinal cord injury.

For peer review only

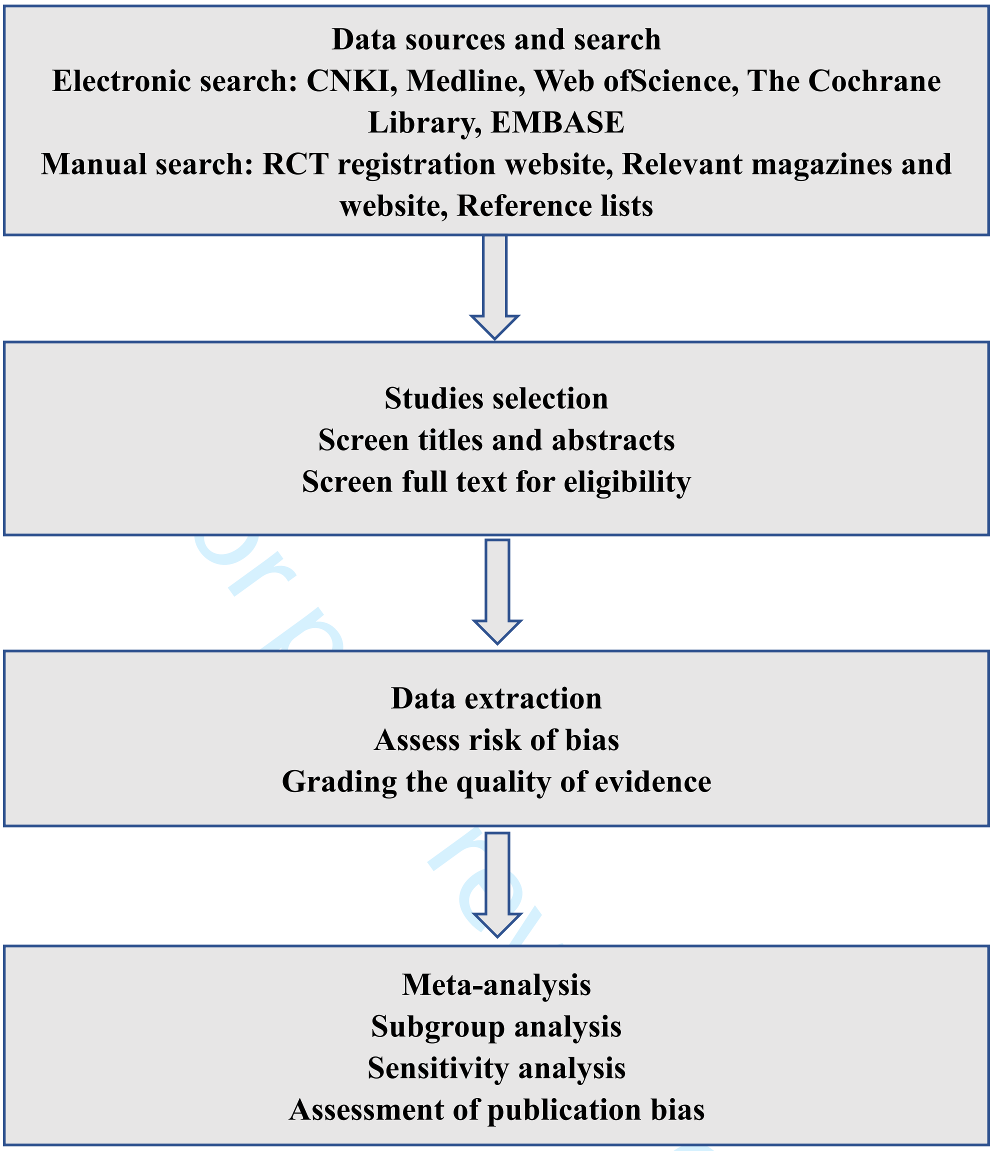

For peer review only

BMJ Open

Effect of robotic-assisted gait training on gait and motor function in spinal cord injury: a protocol of a systematic review with meta-analysis

Journal:	*BMJ Open*
Manuscript ID	bmjopen-2022-070675.R1
Article Type:	Protocol
Date Submitted by the Author:	17-Apr-2023
Complete List of Authors:	wang, lei; Hunan Provincial People's Hospital,<br>peng, lin; Tongji Hospital of Tongji Medical College of Huazhong University of Science and Technology<br>chen, lian; Hunan Provincial People's Hospital
**Primary Subject Heading**:	Rehabilitation medicine
Secondary Subject Heading:	Rehabilitation medicine, Neurology
Keywords:	REHABILITATION MEDICINE, NEUROSURGERY, Neurological injury < NEUROLOGY

SCHOLARONE™
Manuscripts

Effect of robotic-assisted gait training on gait and motor function in spinal cord injury: a protocol of a systematic review with meta-analysis

First author:

Lei Wang, wangleipx168@163.com, Department of Rehabilitation Medicine, Hunan Provincial People's Hospital (The First Affiliated Hospital of Hunan Normal University), Changsha 410005, Hunan, Peoples R China

lin-jin Peng，pjlkim@163.com, Tongji Hospital, Tongji Medical College, Huazhong University of Science & Technology, Wuhan 430000, Hubei Province, China.

Corresponding Author:

lian-ai Chen, 652326303@qq.com , Department of Rehabilitation Medicine, Hunan Provincial People's Hospital (The First Affiliated Hospital of Hunan Normal University), Changsha 410005, Hunan, Peoples R China.

For peer review only

For peer review only - http://bmjopen.bmj.com/site/about/guidelines.xhtml

Effect of robotic-assisted gait training on gait and motor function in spinal cord injury: a protocol of a systematic review with meta-analysis

Abstract：

Introduction: Robotic-assisted gait training (RAGT) has been reported to be effective in the rehabilitation of patients with spinal cord injury (SCI). However, studies on RAGT showed different results because they varied in terms of the number of samples. Thus, summarizing studies based on robotic-related factors is critical for the accurate estimation of the effects of RAGT on SCI. This work aims to search for strong evidence showing that using RAGT is effective in the treatment of SCI and to analyze the deficiencies of current studies.

Methods and analysis: The following publication databases were electronically searched in December 2022 without restrictions on publication year: Medline, Cochrane Library, Web of Science, Embase, PubMed, the Cochrane Central Register of Controlled Trials, and China National Knowledge Infrastructure. All articles on randomized controlled trials using RAGT to treat SCI that were published in English and Chinese and met the following criteria will be included. Outcomes included motor function, and gait parameters included those assessed by using instrumented gait assessment, the Berg balance scale, the 10 m walking speed test, the 6 min walking endurance test, the functional ambulation category scale, the Walking index of SCI, and the ASIA assessment scale. Research selection, data extraction, and quality assessment will be conducted independently by two reviewers to ensure that all relevant studies are free from personal bias. The Cochrane Bias Risk Assessment Tool will be used to assess the risk of bias. Review Manager V.5.3 software will be utilized to produce deviation risk maps and perform paired meta-analyses.

Strengths and limitations of this study

For peer review only

For peer review only - http://bmjopen.bmj.com/site/about/guidelines.xhtml

1.This study will be the first meta-analysis to systematically evaluate the efficacy and safety of RAGT in the treatment of SCI.

2.The results of this study will provide evidence for the treatment of SCI patients, and help therapists and patients to choose appropriate treatment methods.

3.To ensure that all relevant studies are free from personal bias, two reviewers will independently conduct research selection, data extraction and quality assessment.

4.The language categories of the research search are only included in English and Chinese, and the final search results will have some bias.

Key words: Spinal Cord Injuries; Motor disorders; Rehabilitation; Robotics, Gait Analysis

Ethics and dissemination: Ethics approval is not required for systematic reviews and network meta-analyses. The results will be submitted to a peer-reviewed journal or presented at a conference.

Trial registration number: PROSPERO (CRD42022319555).

Introduction

Spinal cord injury (SCI) is a serious disabling disease that often causes paraplegia or quadriplegia and affects the patient's sensory, motor, and autonomic nervous functions[1, 2]. SCI leads to a variety of complications, such as pressure ulcers, lung infections, and urinary tract infections[3]. It affects the quality of life and living standard of patients and imposes a heavy burden on families[4] and society. It ultimately shortens the life expectancy of patients[5]. In addition, the mortality rate of patients with spinal cord injury is higher than that of the general population [6-8]. National statistical data show that the incidence rate of SCI is increasing annually and that the incidence rate of SCI per million residents is 9.3 persons/year[9]. During the rehabilitation treatment of SCI, improving the walking ability, self-care ability, and self-esteem of patients is an important aspect that helps

For peer review only

For peer review only - http://bmjopen.bmj.com/site/about/guidelines.xhtml

patients return to society and reduces their costs. Therefore, increased exercise capacity of the lower limbs is crucial to daily independence and social reintegration for this population, which mainly function in standing and walking[10, 11].

Robot-assisted gait training (RAGT) can improve the walking ability[12], lower limb strength, and independence of patients with incomplete SCI[13]. It can also improve balance function[14]. RAGT has been gradually applied in patients with SCI. Some clinical evidence shows that in patients with SCI, robots for lower limb rehabilitation can effectively and safely improve walking ability; reduce pressure ulcers[15], lung infections[8], urinary tract infections, and other complications[16]; improve dignity; and reduce costs. However, high-quality evidence-based medical studies that systematically evaluated the efficacy of RAGT in the treatment of SCI remain scarce.

Summarizing studies based on RAGT-related factors is critical for the accurate estimation of the effects of RAGT on SCI. This meta-analysis aims to evaluate systematically the efficacy of RAGT in alleviating motor dysfunction and restoring speech ability in patients with SCI according to randomized clinical trials (RCTs); find strong evidence demonstrating that using RAGT is effective in the treatment of SCI; and analyze the deficiencies of current studies.

Methods

The protocol of this systematic review was planned and conducted in accordance with the Preferred Reporting Items for Systematic Reviews and Meta-Analyses Protocols Guideline [17] and PRISMA 2020 guidelines [18] and was performed following a protocol registered in PROSPERO (CRD42022319555). The plan starts on March 1, 2023 and ends on June 1.The review process is shown in Figure

Search strategy

For peer review only - http://bmjopen.bmj.com/site/about/guidelines.xhtml

Two reviewers (Jin-lin Peng and Lei Wang) electronically searched the following publication databases in December 2022 without restrictions on publication year: Medline, Cochrane Library, Web of Science, Embase, PubMed, the Cochrane Central Register of Controlled Trials, and China National Knowledge Infrastructure. Various combinations of keywords, including "motor disorders," "robotics", "robotic assisted gait training," "noninvasive brain stimulation," "SCI", and "gait analysis", were used as search terms. The key terms matched the appropriate Medical Subject Headings terms. Presearches were performed. Then, the final search was conducted follows: Relevant journals and references of review articles were manually searched online to identify papers that may have been missed in the electronic database searches.

Eligibility criteria

Inclusion criteria

(1) Study design: Only RCTs were included. (2) Selected population: Participants diagnosed with SCI, namely, individuals with any level of traumatic SCI, regardless of the time since injury, sex, and age, were included. (3) Type of intervention: The experimental groups received RAGT or RAGT combined with other physical therapies. The control group not received RAGT or received other types of physical therapy. (4) Comparison: The treated subjects were compared at baseline then with the control or sham-stimulated subjects. (5) Type of outcomes measured: Gait analysis indicators, including gait speed (m/s), step length (cm), double support phase (% walking cycle), single support phase (% walking cycle), and symmetry index; Berg balance scale; ASIA assessment scale; Holden walking ability classification (functional ambulation category scale); 10 m walking speed test; 6 min walking endurance test; and WISCI Ⅱ score.

Exclusion criteria

For peer review only - http://bmjopen.bmj.com/site/about/guidelines.xhtml

Studies involving animal research, conference research, protocol studies, or computer model research and duplicate papers were excluded. Two reviewers (Jin-lin Peng and Lei Wang) independently screened titles and abstracts to identify articles reporting studies that met the inclusion criteria. Then, the full-text versions of the identified articles were obtained and separately screened to ensure that they met the inclusion criteria. A third reviewer (Ai-lian Chen) made the final assessment regarding whether or not full-text papers met the inclusion criteria.

Data extraction

A reviewer (Lei Wang) prepared the general information and data collection process by another reviewer (Jin-lin Peng). The format of data collection included the following factors: research design, participants (number, diagnosis, age, and target population numbers in each group), eligibility criteria, intervention used on the research group and control group (i.e., site of stimulation, intensity, number of sessions, and time of each session), and outcomes of interest.

Quality assessment

The quality evaluation of the included studies was performed independently by two reviewers (Jin-lin Peng and Lei Wang) and was revised by the third reviewer (Ai-lian Chen). The methodological quality of the intervention studies was assessed by using the Physiotherapy Evidence Database (PEDro) scale. The PEDro scale is a valid and reliable measure of the methodological quality of RCTs. This 10-item scale is based on the core criteria for RCT quality assessment[19]. The quality of papers was classified as follows in accordance with the PEDro scale: Studies with scores of less than 6 points were considered low-quality studies, whereas those with scores equal to or greater than 6 points were considered high-quality studies (where scores of 6–7 indicate good quality and those of 8–10 indicate excellent quality)[20].

For peer review only - http://bmjopen.bmj.com/site/about/guidelines.xhtml

The GRADEpro GDT online tool was used to evaluate the level of evidence quality of the outcome indicators. The tool is available at its official website http://www.guidelinedevelopment.org/. The GRADEpro GDT online tool for evaluating the quality of outcome indicators includes five degrading factors: risk of bias, inconsistency, indirectness, imprecision, and other considerations.[21] The quality of evidence can be divided into four levels of "high", "moderate", "low", and "very low."[22]

Risk-of-bias assessment of individual studies

The quality of the included studies was evaluated and their scores were compared in a consensus meeting between two independent authors (Jin-lin Peng and Lei Wang) to minimize errors and potential biases in the evaluation. In the event of any disagreement, a third author (Ai-lian Chen) was included in the discussion for a final consensus. The Cochrane Risk of Bias 2.0 tool[23] was used to assess the risk of bias of the articles. Each article was assessed for selection bias (random sequence generation and allocation concealment), performance bias (blinding of participants and personnel), detection bias (blinding of outcome assessment), attrition bias (incomplete outcome data reporting), and reporting bias (selective outcome reporting). Each domain was rated as high risk of bias, unclear of bias, or low risk of bias. The risk map of the biases of the studies' quality was prepared with RevMan 5.2 software.

Patient and public Involvement

No patient participated in writing the system review plan. However, the results will be disseminated to patients with SCI.

Statistical analysis

A meta-analysis will be conducted by using Review Manager 5.3. Heterogeneity between studies

For peer review only - http://bmjopen.bmj.com/site/about/guidelines.xhtml

will be evaluated on the basis of the I^2 statistic for the quantification of the proportion of the total outcome attributable to variability among studies. The following ranges were defined: I^2 = 0%–30% (no heterogeneity), I^2 = 30%–49% (moderate heterogeneity), I^2 = 50%–74% (substantial heterogeneity), and I^2 = 75%–100% (considerable heterogeneity)[24]. On the basis of heterogeneity, a random-effects model was used when I^2 > 30%, and a fixed-effects model was utilized when I^2 = 0%–30%.

For the comparison of data from different scales, pooled statistics will be calculated by using standardized mean differences (SMDs). Means and standard deviations after intervention and follow-up evaluation for the RAGT and control groups (when relevant) will be applied to compute SMDs.

Addressing missing data

The original author will be contacted for additional information regarding missing data. In the absence of a reply, the data will be calculated on the basis of the availability factor. The potential effect of the missing data on meta-analysis results will be tested through sensitivity analysis.

Subgroup analysis

Grouping analysis will be performed to address potential heterogeneity and inconsistencies and will be conducted in accordance with age, gender, SCI plane, disease course, treatment prescription, and treatment duration. At the same time, meta-analysis will be conducted to explore the possible sources of heterogeneity.

Sensitivity analysis

For the verification of the robustness of the research conclusion, sensitivity analysis will be conducted on the main results to assess the effect of method quality, research quality, sample size,

For peer review only - http://bmjopen.bmj.com/site/about/guidelines.xhtml

missing data, and analysis methods on the results of this review[25].

Assessment of publication bias

Each included study will be evaluated in accordance with the PEDro scale. Funnel charts will be used to assess the publication bias of the main results included in the study. If the funnel chart is found to be asymmetrical, attempts will be made to explain its asymmetry [26].

Discussion

RAGT can improve the walking ability of patients with incomplete SCI and can be used by patients with stable vital signs. For patients with complete SCI, RAGT acts mainly to maintain the range of motion of joints. In recent years, studies on using RAGT to improve walking ability in SCI have increased, and the new exoskeleton robot for lower limb rehabilitation has shown the advantage of safe transfer. Our current query shows that our work is the first systematic review and meta-analysis on RAGT for patients with SCI. The results of this meta-analysis can help patients and therapists select the appropriate treatment method for SCI and improve new options on the basis of the comparative evidence for effectiveness and safety. We hope that the results of this study will provide evidence for guideline recommendations.

Data Availability

The datasets used and analyzed in the current study are included in this article.

Ethical Approval

This research is a review, does not involve ethical issues, and did not apply for ethical approval.

Funding

This study has no funding support.

Disclosure

All authors have read and approved the final manuscript.

For peer review only - http://bmjopen.bmj.com/site/about/guidelines.xhtml

Contributors

WL and P-JL, as the first authors, have made equal contributions to this work. Research concept and design: WL and C-AL. Data acquisition: WL and P-JL. Draft: WL and P-JL. Supervised by: C-AL. All the authors approved the publication of the Protocol.

Conflicts of Interest

All authors declare no potential conflicts of interest with respect to the research, authorship, and/or publication of this study.

参考文献

[1] Eckert MJ, Martin MJ. Trauma: Spinal Cord Injury[J]. Surg Clin North Am, 2017,97(5):1031-1045. DOI: 10.1016/j.suc.2017.06.008.

[2] Anjum A, Yazid MD, Fauzi Daud M, et al. Spinal Cord Injury: Pathophysiology, Multimolecular Interactions, and Underlying Recovery Mechanisms[J]. Int J Mol Sci, 2020,21(20)DOI: 10.3390/ijms21207533.

[3] Stricsek G, Ghobrial G, Wilson J, et al. Complications in the Management of Patients with Spine Trauma[J]. Neurosurg Clin N Am, 2017,28(1):147-155. DOI: 10.1016/j.nec.2016.08.007.

[4] Zhang JM, Li N, Zhu L, et al. Effects of pelvic floor biofeedback electrical stimulation combined with lower limb rehabilitation robot training on intestinal function of patients with spinal cord injury [J]. Journal of Brain and Nervous Diseases,2021,29(01):53-57.

[5] Xiang XN, Zhong HY, He HC. Research progress of lower limb exoskeleton rehabilitation robot in improving walking ability of patients with spinal cord injury [J]. Chinese Journal of Rehabilitation Medicine,2020,35(01):119-122. DOI：CNKI:SUN:ZGKF.0.2020-01-024

[6] Buzzell A, Chamberlain JD, Eriks-Hoogland I, et al. All-cause and cause-specific mortality following non-traumatic spinal cord injury: evidence from a population-based cohort study in Switzerland[J]. Spinal Cord, 2020,58(2):157-164. DOI: 10.1038/s41393-019-0361-6.

[7] Mirzaeva L, Lobzin S, Tcinzerling N, et al. Complications and mortality after acute traumatic spinal cord injury in Saint Petersburg, Russia[J]. Spinal Cord, 2020,58(9):970-979. DOI: 10.1038/s41393-020-0458-y.

[8] Li R, Ding M, Wang J, et al. Effectiveness of robotic-assisted gait training on cardiopulmonary fitness and exercise capacity for incomplete spinal cord injury: A systematic review and meta-analysis of randomized controlled trials[J]. Clin Rehabil, 2023,37(3):312-329. DOI: 10.1177/02692155221133474.

[9] Bárbara-Bataller E, Méndez-Suárez JL, Alemán-Sánchez C, et al. Change in the profile of traumatic spinal cord injury over 15 years in Spain[J]. Scand J Trauma Resusc Emerg Med, 2018,26(1):27. DOI: 10.1186/s13049-018-0491-4.

[10] Mahooti F, Raheb G, Alipour F, et al. Psychosocial challenges of social reintegration for people with spinal cord injury: a qualitative study[J]. Spinal Cord, 2020,58(10):1119-1127. DOI: 10.1038/s41393-020-0449-z.

[11] Rahimi M, Torkaman G, Ghabaee M, et al. Advanced weight-bearing mat exercises combined with

For peer review only - http://bmjopen.bmj.com/site/about/guidelines.xhtml

functional electrical stimulation to improve the ability of wheelchair-dependent people with spinal cord injury to transfer and attain independence in activities of daily living: a randomized controlled trial[J]. Spinal Cord, 2020,58(1):78-85. DOI: 10.1038/s41393-019-0328-7.

[12] Grasmücke D, Zieriacks A, Jansen O, et al. Against the odds: what to expect in rehabilitation of chronic spinal cord injury with a neurologically controlled Hybrid Assistive Limb exoskeleton. A subgroup analysis of 55 patients according to age and lesion level[J]. Neurosurg Focus, 2017,42(5):E15. DOI: 10.3171/2017.2.FOCUS171.

[13] Holanda LJ, Silva P, Amorim TC, et al. Robotic assisted gait as a tool for rehabilitation of individuals with spinal cord injury: a systematic review[J]. J Neuroeng Rehabil, 2017,14(1):126. DOI: 10.1186/s12984-017-0338-7.

[14] Nam KY, Kim HJ, Kwon BS, et al. Robot-assisted gait training (Lokomat) improves walking function and activity in people with spinal cord injury: a systematic review[J]. J Neuroeng Rehabil, 2017,14(1):24. DOI: 10.1186/s12984-017-0232-3.

[15] Rathore A, Wilcox M, Ramirez DZ, et al. Quantifying the human-robot interaction forces between a lower limb exoskeleton and healthy users[J]. Annu Int Conf IEEE Eng Med Biol Soc, 2016,2016:586-589. DOI: 10.1109/EMBC.2016.7590770.

[16] Pattanakuhar S, Ahmedy F, Setiono S, et al. Impacts of Bladder Managements and Urinary Complications on Quality of Life: Cross-sectional Perspectives of Persons With Spinal Cord Injury Living in Malaysia, Indonesia, and Thailand[J]. Am J Phys Med Rehabil, 2023,102(3):214-221. DOI: 10.1097/PHM.0000000000002066.

[17] Cumpston M, Li T, Page MJ, et al. Updated guidance for trusted systematic reviews: a new edition of the Cochrane Handbook for Systematic Reviews of Interventions. Cochrane Database Syst Rev. 2019. 10: ED000142.

[18] Page MJ, McKenzie JE, Bossuyt PM, et al. The PRISMA 2020 statement: an updated guideline for reporting systematic reviews[J]. BMJ, 2021,372:n71. DOI: 10.1136/bmj.n71.

[19] Elbanna ST, Elshennawy S, Ayad MN. Noninvasive Brain Stimulation for Rehabilitation of Pediatric Motor Disorders Following Brain Injury: Systematic Review of Randomized Controlled Trials. Arch Phys Med Rehabil. 2019. 100(10): 1945-1963.

[20] Maher CG, Sherrington C, Herbert RD, Moseley AM, Elkins M. Reliability of the PEDro scale for rating quality of randomized controlled trials. Phys Ther. 2003. 83(8): 713-21.

[21] Nasser M, Fedorowicz Z. Grading the quality of evidence and strength of recommendations: the GRADE approach to improving dental clinical guidelines[J]. J Appl Oral Sci, 2011,19(1):0. DOI: 10.1590/s1678-77572011000100001.

[22] Brożek JL, Akl EA, Compalati E, et al. Grading quality of evidence and strength of recommendations in clinical practice guidelines part 3 of 3. The GRADE approach to developing recommendations[J]. Allergy, 2011,66(5):588-595. DOI: 10.1111/j.1398-9995.2010.02530.x.

[23] Sterne J, Savović J, Page MJ, et al. RoB 2: a revised tool for assessing risk of bias in randomised trials[J]. BMJ, 2019,366:l4898. DOI: 10.1136/bmj.l4898.

[24] Higgins JP, Thompson SG. Quantifying heterogeneity in a meta-analysis[J]. Stat Med, 2002,21(11):1539-1558. DOI: 10.1002/sim.1186.

[25] Li J, Zhong D, Ye J, et al. Rehabilitation for balance impairment in patients after stroke: a protocol of a systematic review and network meta-analysis[J]. BMJ Open, 2019,9(7):e026844. DOI: 10.1136/bmjopen-2018-026844.

[26] Sterne JA, Sutton AJ, Ioannidis JP, et al. Recommendations for examining and interpreting funnel plot

For peer review only - http://bmjopen.bmj.com/site/about/guidelines.xhtml

asymmetry in meta-analyses of randomised controlled trials[J]. BMJ, 2011,343:d4002. DOI: 10.1136/bmj.d4002.

Figure: Flow chart of meta-analysis for robotic-assisted gait training in patients with spinal cord injury.

For peer review only

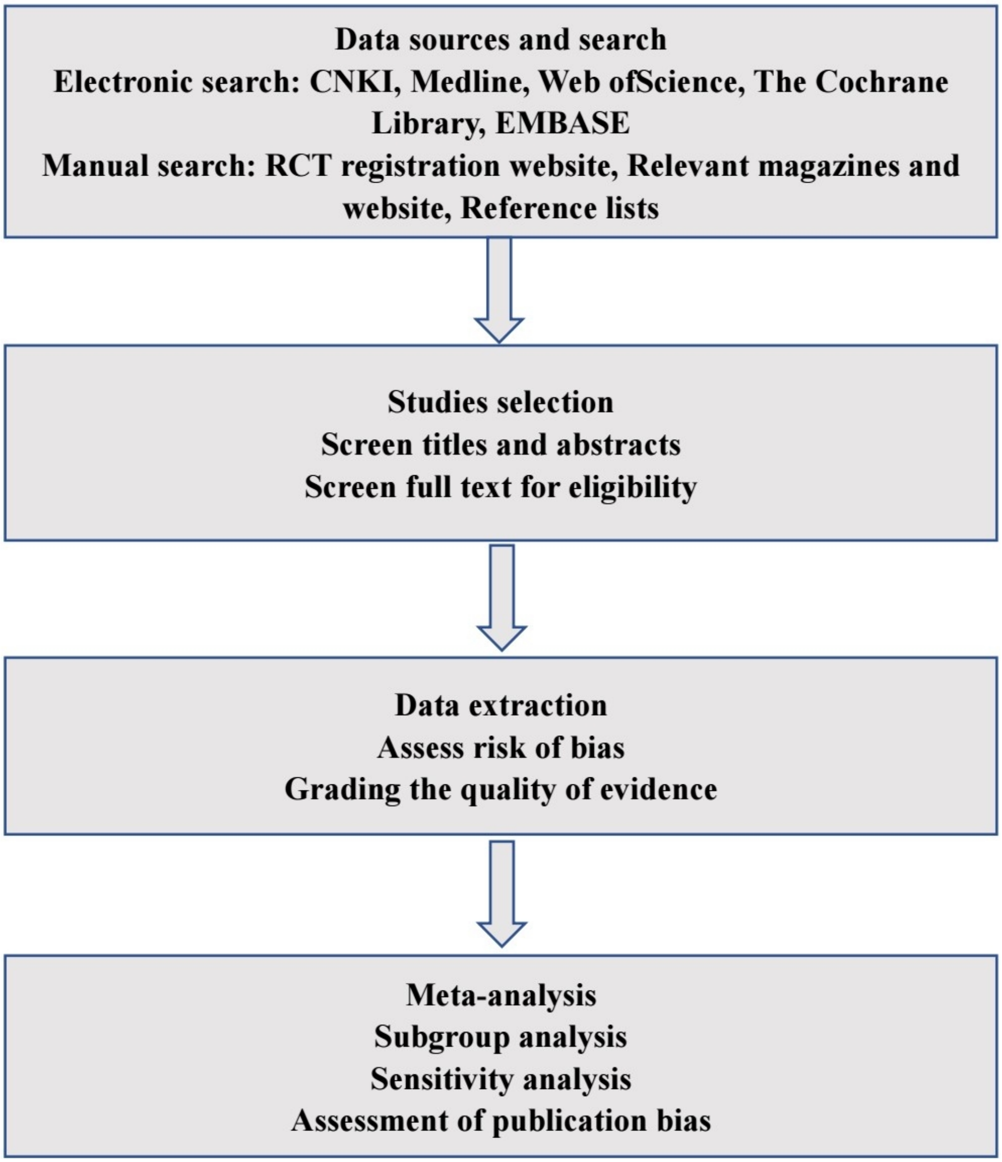

262x303mm (96 x 96 DPI)

BMJ Open

Effect of robotic-assisted gait training on gait and motor function in spinal cord injury: a protocol of a systematic review with meta-analysis

Journal:	*BMJ Open*
Manuscript ID	bmjopen-2022-070675.R2
Article Type:	Protocol
Date Submitted by the Author:	05-Jun-2023
Complete List of Authors:	wang, lei; Hunan Provincial People's Hospital,<br>peng, lin; Tongji Hospital of Tongji Medical College of Huazhong University of Science and Technology<br>chen, lian; Hunan Provincial People's Hospital
**Primary Subject Heading**:	Rehabilitation medicine
Secondary Subject Heading:	Rehabilitation medicine, Neurology
Keywords:	REHABILITATION MEDICINE, NEUROSURGERY, Neurological injury < NEUROLOGY

SCHOLARONE™
Manuscripts

Effect of robotic-assisted gait training on gait and motor function in spinal cord injury: a protocol of a systematic review with meta-analysis

First author:

Lei Wang, wangleipx168@163.com, Department of Rehabilitation Medicine, Hunan Provincial People's Hospital (The First Affiliated Hospital of Hunan Normal University), Changsha 410005, Hunan, Peoples R China

Jin-lin Peng(lin-jin Peng)，pjlkim@163.com, Tongji Hospital, Tongji Medical College, Huazhong University of Science & Technology, Wuhan 430000, Hubei Province, China.

Corresponding Author:

Ai-lian Chen (lian-ai Chen), 652326303@qq.com , Department of Rehabilitation Medicine, Hunan Provincial People's Hospital (The First Affiliated Hospital of Hunan Normal University), Changsha 410005, Hunan, Peoples R China.

(Note: The name in the article is correct, and the name in parentheses comes from ScholarOne. As ScholarOne cannot modify the name, it is hereby stated in the article)

For peer review only

For peer review only - http://bmjopen.bmj.com/site/about/guidelines.xhtml

Effect of robotic-assisted gait training on gait and motor function in spinal cord injury: a protocol of a systematic review with meta-analysis

Abstract：

Introduction: Robotic-assisted gait training (RAGT) has been reported to be effective in rehabilitating patients with spinal cord injury (SCI). However, studies on RAGT showed different results due to a varied number of samples. Thus, summarising studies based on robotic-related factors is critical for the accurate estimation of the effects of RAGT on SCI. This work aims to search for strong evidence showing that using RAGT is effective in treating SCI and analyse the deficiencies of current studies.

Methods and analysis: The following publication databases were electronically searched in December 2022 without restrictions on publication year: Medline, Cochrane Library, Web of Science, Embase, PubMed, the Cochrane Central Register of Controlled Trials and China National Knowledge Infrastructure. Various combinations of keywords, including 'motor disorders', 'robotics', 'robotic-assisted gait training', 'Spinal Cord Injuries', 'SCI' and 'gait analysis' were used as search terms. All articles on randomised controlled trials (excluding retrospective trials) using RAGT to treat SCI that were published in English and Chinese and met the inclusion criteria were included. Outcomes included motor function, and gait parameters included those assessed by using the instrumented gait assessment, the Berg balance scale, the 10-m walk speed test, the 6-min walk endurance test, the functional ambulation category scale, the Walking index of SCI and the ASIA assessment scale. Research selection, data extraction and quality assessment were conducted independently by two reviewers to ensure that all relevant studies were free from personal bias. In

For peer review only - http://bmjopen.bmj.com/site/about/guidelines.xhtml

addition, the Cochrane Bias Risk Assessment Tool was used to assess the risk of bias. Review Manager V.5.3 software was utilised to produce deviation risk maps and perform paired meta-analyses.

Strengths and limitations of this study

1. This study was the first meta-analysis to systematically evaluate the efficacy and safety of RAGT in the treatment of SCI.

2. The results of this study provided evidence for the treatment of SCI patients and helped therapists and patients to choose appropriate treatment methods.

3. Two reviewers independently conducted research selection, data extraction and quality assessment to ensure that all relevant studies were free from personal bias.

4. The language categories of the research search were only included in English and Chinese, and the final search results would have some bias.

Key words: Spinal Cord Injuries; Motor disorders; Rehabilitation; Robotics, Gait Analysis

Ethics and dissemination: Ethics approval is not required for systematic reviews and network meta-analyses. The results will be submitted to a peer-reviewed journal or presented at a conference.

Trial registration number: PROSPERO (CRD42022319555).

Introduction

Spinal cord injury (SCI) is a serious disabling disease that often causes paraplegia or quadriplegia and affects patient's sensory, motor and autonomic nervous functions[1, 2]. SCI leads to various complications, such as pressure ulcers, lung infections and urinary tract infections[3]. It also affects patients' quality of life and living standard and imposes a heavy burden on families[4] and society. It ultimately shortens patients' life expectancy [5]. In addition, the mortality rate of patients with SCI

For peer review only - http://bmjopen.bmj.com/site/about/guidelines.xhtml

is higher than that of the general population [6–8]. National statistical data show an increasing incidence rate of SCI annually, and that the incidence rate of SCI per million residents is 9.3 persons/year[9]. During the rehabilitation treatment of SCI, improving the walking ability, self-care ability and self-esteem of patients is an important aspect that helps them return to society and reduces their costs. Therefore, increased exercise capacity of the lower limbs is crucial to daily independence and social reintegration for this population, which mainly functions in standing and walking[10, 11].

Robot-assisted gait training (RAGT) can improve the walking ability[12], lower limb strength and independence of patients with incomplete SCI[13]. RAGT can also improve balance function[14] and has been gradually applied in patients with SCI. In patients with SCI, robots for lower limb rehabilitation can effectively and safely improve walking ability; reduce pressure ulcers[15], lung infections[8], urinary tract infections and other complications[16]; improve dignity; and reduce costs. However, high-quality evidence-based medical studies that systematically evaluated the efficacy of RAGT in the treatment of SCI remain scarce.

Therefore, summarising studies based on RAGT-related factors is critical for the accurate estimation of the effects of RAGT on SCI. This meta-analysis aims to systematically evaluate the efficacy of RAGT in alleviating motor dysfunction and restoring speech ability in patients with SCI based on randomised clinical trials (RCTs), find strong evidence demonstrating that using RAGT is effective in the treatment of SCI and analyse the deficiencies of current studies.

Methods

The protocol of this systematic review was planned and conducted following the Preferred Reporting Items for Systematic Reviews and Meta-Analyses Protocols Guideline [17] and PRISMA

For peer review only

For peer review only - http://bmjopen.bmj.com/site/about/guidelines.xhtml

2020 guidelines [18] and was performed following a protocol registered in PROSPERO (CRD42022319555). The plan starts on March 1, 2023 and ends on June 1. The review process is shown in Figure 1.

Search strategy

Two reviewers (Jin-lin Peng and Lei Wang) electronically searched the following publication databases in December 2022 without restrictions on publication year: Medline, Cochrane Library, Web of Science, Embase, PubMed, the Cochrane Central Register of Controlled Trials and China National Knowledge Infrastructure. Various combinations of keywords, including 'motor disorders', 'robotics', 'robotic-assisted gait training', 'Spinal Cord Injuries', 'SCI' and 'gait analysis' were used as search terms. The key terms matched the appropriate Medical Subject Heading terms. Presearches were performed. Then, the final search was conducted, relevant journals and references of review articles were manually searched online to identify papers that may have been missed in the electronic database searches.

Eligibility criteria

Inclusion criteria

(1) Study design: Only RCTs were included. (2) Selected population: Participants diagnosed with SCI, namely, individuals with any level of traumatic SCI, regardless of the time since injury, sex and age were included. (3) Type of intervention: The experimental groups received RAGT or RAGT combined with other physical therapies. The control group not received RAGT or received other types of physical therapy. (4) Comparison: The treated subjects were compared at baseline and then with the control or sham-stimulated subjects. (5) Type of outcomes measured: Gait analysis indicators, including gait speed (m/s), step length (cm), double support phase (% walking cycle),

For peer review only

single support phase (% walking cycle) and symmetry index; Berg balance scale; ASIA assessment scale; Holden walking ability classification (functional ambulation category scale); 10-m walk speed test; 6-min walk endurance test; and WISCI Ⅱ score.

Exclusion criteria

Studies involving animal research, conference research, protocol studies or computer model research and duplicate papers were excluded. Two reviewers (Jin-lin Peng and Lei Wang) independently screened titles and abstracts to identify articles reporting studies that met the inclusion criteria. Then, the full-text versions of the identified articles were obtained and separately screened to ensure that they met the inclusion criteria. Moreover, a third reviewer (Ai-lian Chen) made the final assessment regarding whether or not full-text papers met the inclusion criteria.

Data extraction

A reviewer (Lei Wang) prepared the general information and data collection process by another reviewer (Jin-lin Peng). The format of data collection included research design, participants (number, diagnosis, age and target population numbers in each group), eligibility criteria, intervention used on the research group and control group (i.e. site of stimulation, intensity, number of sessions and time of each session) and outcomes of interest.

Quality assessment

The quality evaluation of the included studies was performed independently by two reviewers (Jin-lin Peng and Lei Wang) and was revised by the third reviewer (Ai-lian Chen). The methodological quality of the intervention studies was assessed using the Physiotherapy Evidence Database (PEDro) scale. The PEDro scale is a valid and reliable measure of the methodological quality of RCTs. This 10-item scale is based on the core criteria for RCT quality assessment[19]. The quality of papers was

For peer review only - http://bmjopen.bmj.com/site/about/guidelines.xhtml

classified based on the PEDro scale. Studies with scores of less than 6 points were considered low-quality studies, whereas those with scores equal to or greater than 6 points were considered high-quality studies (scores of 6–7 indicate good quality and 8–10 indicate excellent quality)[20]. The GRADEpro GDT online tool was used to evaluate the level of evidence quality of the outcome indicators. The tool is available at its official website http://www.guidelinedevelopment.org/. The GRADEpro GDT online tool for evaluating the quality of outcome indicators includes five degrading factors, namely, risk of bias, inconsistency, indirectness, imprecision and other considerations[21]. The quality of evidence can be divided into four levels, namely, 'high', 'moderate', 'low' and 'very low'[22].

Risk-of-bias assessment of individual studies

The quality of the included studies was evaluated and their scores were compared in a consensus meeting between two independent authors (Jin-lin Peng and Lei Wang) to minimise errors and potential biases in the evaluation. However, in the event of any disagreement, a third author (Ai-lian Chen) was included in the discussion for a final consensus. The Cochrane Risk of Bias 2.0 tool[23] was used to assess the articles' risk of bias. Each article was assessed for selection bias (random sequence generation and allocation concealment), performance bias (blinding of participants and personnel), detection bias (blinding of outcome assessment), attrition bias (incomplete outcome data reporting) and reporting bias (selective outcome reporting). Each domain was rated as high risk of bias, unclear of bias or low risk of bias. The risk map of the biases of the studies' quality was prepared with Review Manager 5.3.

Patient and Public Involvement

No patient participated in writing the system review plan. However, the results were disseminated

For peer review only - http://bmjopen.bmj.com/site/about/guidelines.xhtml

to patients with SCI.

Statistical analysis

A meta-analysis was conducted using Review Manager 5.3. Heterogeneity between studies was evaluated based on the I^2 statistic for the quantification of the proportion of the total outcome attributable to variability amongst studies. The following ranges were defined: I^2 = 0%–30% (no heterogeneity), I^2 = 30%–49% (moderate heterogeneity), I^2 = 50%–74% (substantial heterogeneity) and I^2 = 75%–100% (considerable heterogeneity)[24]. Based on heterogeneity, a random-effects model was used when I^2 > 30%, and a fixed-effects model was utilised when I^2 = 0%–30%.

For the comparison of data from different scales, pooled statistics were calculated using standardised mean differences (SMDs). Furthermore, means and standard deviations after intervention and follow-up evaluation for the RAGT and control groups (when relevant) were applied to compute SMDs.

Addressing missing data

Regarding missing data, the original author was contacted for additional information. In the absence of a reply, the data was calculated based on the availability factor. The potential effect of the missing data on meta-analysis results was tested through sensitivity analysis.

Subgroup analysis

Analysis results showed a situation wherein heterogeneity was high and subgroup analysis was required. Grouping analysis was conducted based on age (children, adolescents, middle-aged and elderly), SCI level (cervical, thoracic and lumbar), disease course (recovery and sequelae), treatment prescription and treatment duration to address potential heterogeneity and inconsistency. A meta-analysis was also conducted to explore possible sources of heterogeneity.

For peer review only - http://bmjopen.bmj.com/site/about/guidelines.xhtml

Sensitivity analysis

Sensitivity analysis was conducted on the main results to assess the effect of method quality, research quality, sample size, missing data and analysis methods on the results of this review to verify the robustness of the research conclusion [25].

Assessment of publication bias

Each included study was evaluated based on the PEDro scale. Funnel charts were used to assess the publication bias of the main results included in the study. However, when the funnel chart was asymmetrical, attempts were made to explain its asymmetry [26].

Discussion

RAGT can improve the walking ability of patients with incomplete SCI and can be used by patients with stable vital signs. For patients with complete SCI, RAGT primarily acts to maintain the range of motion of joints. In recent years, there is an increasing number of studies on using RAGT to improve walking ability in SCI, and the new exoskeleton robot for lower limb rehabilitation has shown the advantage of safe transfer. Our current query shows that our work is the first systematic review and meta-analysis on RAGT for patients with SCI. The results of this meta-analysis can help patients and therapists select the appropriate treatment method for SCI and improve new options based on the comparative evidence for effectiveness and safety. Therefore, we hope that the results of this study will provide evidence for guideline recommendations.

Study limitations

Articles published in both Chinese and English were included. Articles in other languages were not included, and their exclusion may affect our research. When incorporating outcome indicators, all data were sourced from scale evaluation and gait analysis instruments. The lack of research results

For peer review only - http://bmjopen.bmj.com/site/about/guidelines.xhtml

on neural mechanisms may have had a certain effect on this study.

Data Availability

The datasets used and analysed in the current study are included in this article.

Ethical Approval

This research is a review, does not involve ethical issues and did not apply for ethical approval.

Funding

This study has no funding support.

Disclosure

All authors have read and approved the final manuscript.

Contributors

As the first authors, WL and P-JL have made equal contributions to this work. WL and C-AL for research concept and design. WL and P-JL are responsible for data acquisition. WL and P-JL made the draft, and C-AL did the supervision. All the authors approved the publication of the Protocol.

Conflicts of Interest

All authors declare no potential conflicts of interest with respect to the research, authorship and/or publication of this study.

References

[1] Eckert MJ, Martin MJ. Trauma: Spinal Cord Injury[J]. Surg Clin North Am, 2017,97(5):1031-1045. DOI: 10.1016/j.suc.2017.06.008.

[2] Anjum A, Yazid MD, Fauzi Daud M, et al. Spinal Cord Injury: Pathophysiology, Multimolecular Interactions, and Underlying Recovery Mechanisms[J]. Int J Mol Sci, 2020,21(20)DOI: 10.3390/ijms21207533.

[3] Stricsek G, Ghobrial G, Wilson J, et al. Complications in the Management of Patients with Spine Trauma[J]. Neurosurg Clin N Am, 2017,28(1):147-155. DOI: 10.1016/j.nec.2016.08.007.

[4] Zhang JM, Li N, Zhu L, et al. Effects of pelvic floor biofeedback electrical stimulation combined with lower limb rehabilitation robot training on intestinal function of patients with spinal cord injury [J]. Journal of Brain and Nervous Diseases,2021,29(01):53-57.

[5] Xiang XN, Zhong HY, He HC. Research progress of lower limb exoskeleton rehabilitation robot in improving walking ability of patients with spinal cord injury [J]. Chinese Journal of Rehabilitation Medicine,2020,35(01):119-122. DOI：CNKI:SUN:ZGKF.0.2020-01-024

For peer review only - http://bmjopen.bmj.com/site/about/guidelines.xhtml

For peer review only

[6] Buzzell A, Chamberlain JD, Eriks-Hoogland I, et al. All-cause and cause-specific mortality following non-traumatic spinal cord injury: evidence from a population-based cohort study in Switzerland[J]. Spinal Cord, 2020,58(2):157-164. DOI: 10.1038/s41393-019-0361-6.

[7] Mirzaeva L, Lobzin S, Tcinzerling N, et al. Complications and mortality after acute traumatic spinal cord injury in Saint Petersburg, Russia[J]. Spinal Cord, 2020,58(9):970-979. DOI: 10.1038/s41393-020-0458-y.

[8] Li R, Ding M, Wang J, et al. Effectiveness of robotic-assisted gait training on cardiopulmonary fitness and exercise capacity for incomplete spinal cord injury: A systematic review and meta-analysis of randomized controlled trials[J]. Clin Rehabil, 2023,37(3):312-329. DOI: 10.1177/02692155221133474.

[9] Bárbara-Bataller E, Méndez-Suárez JL, Alemán-Sánchez C, et al. Change in the profile of traumatic spinal cord injury over 15 years in Spain[J]. Scand J Trauma Resusc Emerg Med, 2018,26(1):27. DOI: 10.1186/s13049-018-0491-4.

[10] Mahooti F, Raheb G, Alipour F, et al. Psychosocial challenges of social reintegration for people with spinal cord injury: a qualitative study[J]. Spinal Cord, 2020,58(10):1119-1127. DOI: 10.1038/s41393-020-0449-z.

[11] Rahimi M, Torkaman G, Ghabaee M, et al. Advanced weight-bearing mat exercises combined with functional electrical stimulation to improve the ability of wheelchair-dependent people with spinal cord injury to transfer and attain independence in activities of daily living: a randomized controlled trial[J]. Spinal Cord, 2020,58(1):78-85. DOI: 10.1038/s41393-019-0328-7.

[12] Grasmücke D, Zieriacks A, Jansen O, et al. Against the odds: what to expect in rehabilitation of chronic spinal cord injury with a neurologically controlled Hybrid Assistive Limb exoskeleton. A subgroup analysis of 55 patients according to age and lesion level[J]. Neurosurg Focus, 2017,42(5):E15. DOI: 10.3171/2017.2.FOCUS171.

[13] Holanda LJ, Silva P, Amorim TC, et al. Robotic assisted gait as a tool for rehabilitation of individuals with spinal cord injury: a systematic review[J]. J Neuroeng Rehabil, 2017,14(1):126. DOI: 10.1186/s12984-017-0338-7.

[14] Nam KY, Kim HJ, Kwon BS, et al. Robot-assisted gait training (Lokomat) improves walking function and activity in people with spinal cord injury: a systematic review[J]. J Neuroeng Rehabil, 2017,14(1):24. DOI: 10.1186/s12984-017-0232-3.

[15] Rathore A, Wilcox M, Ramirez DZ, et al. Quantifying the human-robot interaction forces between a lower limb exoskeleton and healthy users[J]. Annu Int Conf IEEE Eng Med Biol Soc, 2016,2016:586-589. DOI: 10.1109/EMBC.2016.7590770.

[16] Pattanakuhar S, Ahmedy F, Setiono S, et al. Impacts of Bladder Managements and Urinary Complications on Quality of Life: Cross-sectional Perspectives of Persons With Spinal Cord Injury Living in Malaysia, Indonesia, and Thailand[J]. Am J Phys Med Rehabil, 2023,102(3):214-221. DOI: 10.1097/PHM.0000000000002066.

[17] Cumpston M, Li T, Page MJ, et al. Updated guidance for trusted systematic reviews: a new edition of the Cochrane Handbook for Systematic Reviews of Interventions. Cochrane Database Syst Rev. 2019. 10: ED000142.

[18] Page MJ, McKenzie JE, Bossuyt PM, et al. The PRISMA 2020 statement: an updated guideline for reporting systematic reviews[J]. BMJ, 2021,372:n71. DOI: 10.1136/bmj.n71.

[19] Elbanna ST, Elshennawy S, Ayad MN. Noninvasive Brain Stimulation for Rehabilitation of Pediatric Motor Disorders Following Brain Injury: Systematic Review of Randomized Controlled Trials. Arch Phys Med Rehabil. 2019. 100(10): 1945-1963.

[20] Maher CG, Sherrington C, Herbert RD, Moseley AM, Elkins M. Reliability of the PEDro scale for rating quality of randomized controlled trials. Phys Ther. 2003. 83(8): 713-21.

For peer review only - http://bmjopen.bmj.com/site/about/guidelines.xhtml

[21] Nasser M, Fedorowicz Z. Grading the quality of evidence and strength of recommendations: the GRADE approach to improving dental clinical guidelines[J]. J Appl Oral Sci, 2011,19(1):0. DOI: 10.1590/s1678-77572011000100001.

[22] Brożek JL, Akl EA, Compalati E, et al. Grading quality of evidence and strength of recommendations in clinical practice guidelines part 3 of 3. The GRADE approach to developing recommendations[J]. Allergy, 2011,66(5):588-595. DOI: 10.1111/j.1398-9995.2010.02530.x.

[23] Sterne J, Savović J, Page MJ, et al. RoB 2: a revised tool for assessing risk of bias in randomised trials[J]. BMJ, 2019,366:l4898. DOI: 10.1136/bmj.l4898.

[24] Higgins JP, Thompson SG. Quantifying heterogeneity in a meta-analysis[J]. Stat Med, 2002,21(11):1539-1558. DOI: 10.1002/sim.1186.

[25] Li J, Zhong D, Ye J, et al. Rehabilitation for balance impairment in patients after stroke: a protocol of a systematic review and network meta-analysis[J]. BMJ Open, 2019,9(7):e026844. DOI: 10.1136/bmjopen-2018-026844.

[26] Sterne JA, Sutton AJ, Ioannidis JP, et al. Recommendations for examining and interpreting funnel plot asymmetry in meta-analyses of randomised controlled trials[J]. BMJ, 2011,343:d4002. DOI: 10.1136/bmj.d4002.

Figure: Flow chart of meta-analysis for robotic-assisted gait training in patients with spinal cord injury.

For peer review only

For peer review only - http://bmjopen.bmj.com/site/about/guidelines.xhtml

For peer review only

For peer review only - http://bmjopen.bmj.com/site/about/guidelines.xhtml

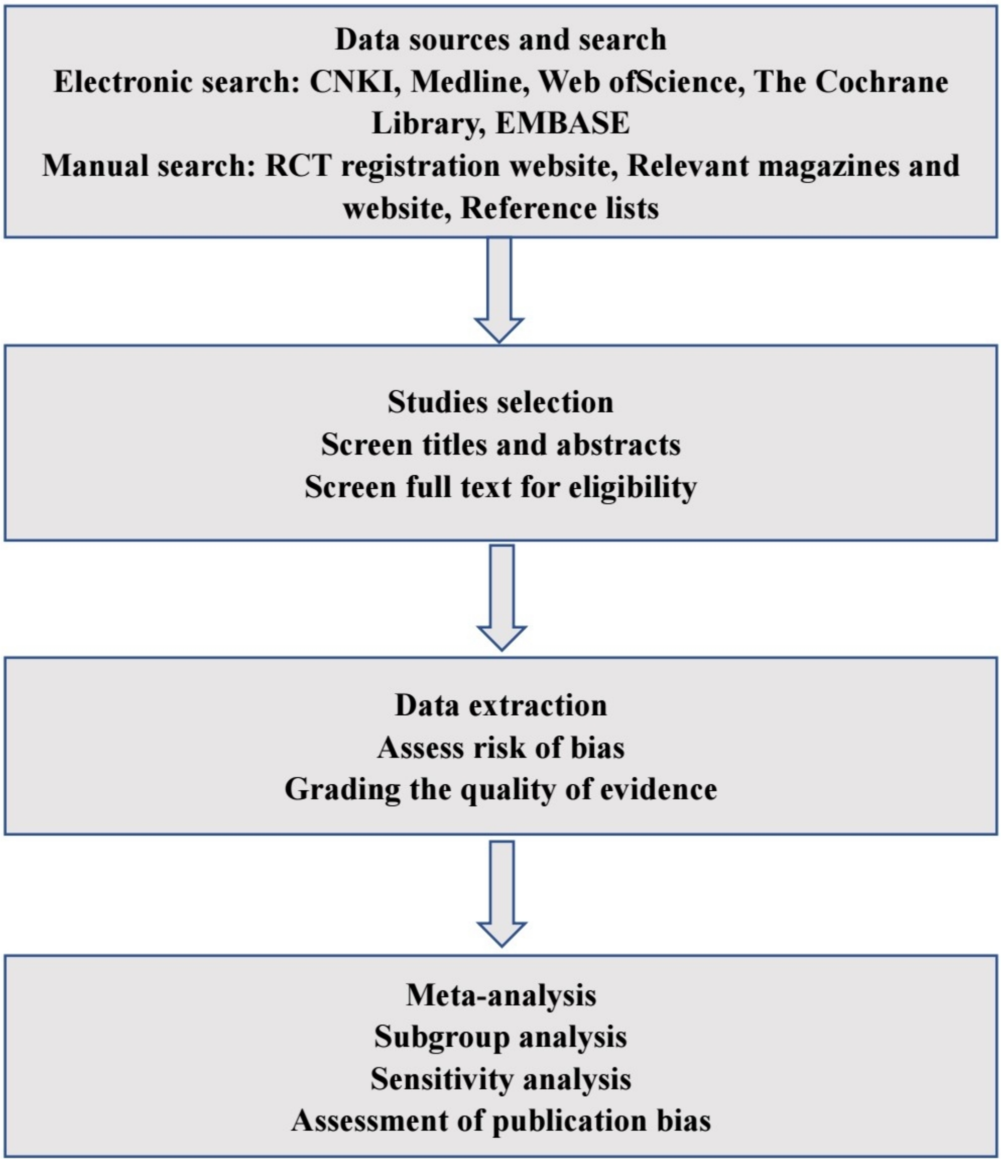

262x303mm (96 x 96 DPI)

PRISMA-P (Preferred Reporting Items for Systematic review and Meta-Analysis Protocols) 2015 checklist: recommended items to address in a systematic review protocol*

Section and topic	Item No	Checklist item	Location where item is reported（line numbers）
**ADMINISTRATIVE INFORMATION**			
Title:			
Identification	1a	Identify the report as a protocol of a systematic review	1
Update	1b	If the protocol is for an update of a previous systematic review, identify as such	
Registration	2	If registered, provide the name of the registry (such as PROSPERO) and registration number	60
Authors:			
Contact	3a	Provide name, institutional affiliation, e-mail address of all protocol authors; provide physical mailing address of corresponding author	4-15
Contributions	3b	Describe contributions of protocol authors and identify the guarantor of the review	212-215
Amendments	4	If the protocol represents an amendment of a previously completed or published protocol, identify as such and list changes; otherwise, state plan for documenting important protocol amendments	
Support:			
Sources	5a	Indicate sources of financial or other support for the review	206-207
Sponsor	5b	Provide name for the review funder and/or sponsor	
Role of sponsor or funder	5c	Describe roles of funder(s), sponsor(s), and/or institution(s), if any, in developing the protocol	
**INTRODUCTION**			**61-85**
Rationale	6	Describe the rationale for the review in the context of what is already known	81-85
Objectives	7	Provide an explicit statement of the question(s) the review will address with reference to participants, interventions, comparators, and outcomes (PICO)	104-113
**METHODS**			
Eligibility criteria	8	Specify the study characteristics (such as PICO, study design, setting, time frame) and report characteristics (such as years considered, language, publication status) to be used as criteria for eligibility for the review	104-113

For peer review only

Information sources	9	Describe all intended information sources (such as electronic databases, contact with study authors, trial registers or other grey literature sources) with planned dates of coverage	92-101
Search strategy	10	Present draft of search strategy to be used for at least one electronic database, including planned limits, such that it could be repeated	92-101
Study records:			
Data management	11a	Describe the mechanism(s) that will be used to manage records and data throughout the review	114-120
Selection process	11b	State the process that will be used for selecting studies (such as two independent reviewers) through each phase of the review (that is, screening, eligibility and inclusion in meta-analysis)	114-120
Data collection process	11c	Describe planned method of extracting data from reports (such as piloting forms, done independently, in duplicate), any processes for obtaining and confirming data from investigators	121-126
Data items	12	List and define all variables for which data will be sought (such as PICO items, funding sources), any pre-planned data assumptions and simplifications	121-126
Outcomes and prioritization	13	List and define all outcomes for which data will be sought, including prioritization of main and additional outcomes, with rationale	121-126
Risk of bias in individual studies	14	Describe anticipated methods for assessing risk of bias of individual studies, including whether this will be done at the outcome or study level, or both; state how this information will be used in data synthesis	142-152
Data synthesis	15a	Describe criteria under which study data will be quantitatively synthesised	121-126
	15b	If data are appropriate for quantitative synthesis, describe planned summary measures, methods of handling data and methods of combining data from studies, including any planned exploration of consistency (such as I^2, Kendall's τ)	156-166
	15c	Describe any proposed additional analyses (such as sensitivity or subgroup analyses, meta-regression)	171-180
	15d	If quantitative synthesis is not appropriate, describe the type of summary planned	167-170
Meta-bias(es)	16	Specify any planned assessment of meta-bias(es) (such as publication bias across studies, selective reporting within studies)	181-184
Confidence in cumulative evidence	17	Describe how the strength of the body of evidence will be assessed (such as GRADE)	181-184

*** It is strongly recommended that this checklist be read in conjunction with the PRISMA-P Explanation and Elaboration (cite when available) for important clarification on the items. Amendments to a review protocol should be tracked and dated. The copyright for PRISMA-P (including checklist) is held by the PRISMA-P Group and is distributed under a Creative Commons Attribution Licence 4.0.**

For peer review only

From: Shamseer L, Moher D, Clarke M, Ghersi D, Liberati A, Petticrew M, Shekelle P, Stewart L, PRISMA-P Group. Preferred reporting items for systematic review and meta-analysis protocols (PRISMA-P) 2015: elaboration and explanation. BMJ. 2015 Jan 2;349(jan02 1):g7647.

For peer review only

BMJ Open

Effect of robotic-assisted gait training on gait and motor function in spinal cord injury: a protocol of a systematic review with meta-analysis

Journal:	*BMJ Open*
Manuscript ID	bmjopen-2022-070675.R3
Article Type:	Protocol
Date Submitted by the Author:	07-Aug-2023
Complete List of Authors:	wang, lei; Hunan Provincial People's Hospital,<br>peng, lin; Tongji Hospital of Tongji Medical College of Huazhong University of Science and Technology<br>chen, lian; Hunan Provincial People's Hospital
**Primary Subject Heading**:	Rehabilitation medicine
Secondary Subject Heading:	Rehabilitation medicine, Neurology
Keywords:	REHABILITATION MEDICINE, NEUROSURGERY, Neurological injury < NEUROLOGY

SCHOLARONE™
Manuscripts

Effect of robotic-assisted gait training on gait and motor function in spinal cord injury: a protocol of a systematic review with meta-analysis

First author:

Lei Wang, wangleipx168@163.com, Department of Rehabilitation Medicine, Hunan Provincial People's Hospital (The First Affiliated Hospital of Hunan Normal University), Changsha 410005, Hunan, Peoples R China

Jin-lin Peng(lin-jin Peng), pjlkim@163.com, Tongji Hospital, Tongji Medical College, Huazhong University of Science & Technology, Wuhan 430000, Hubei Province, China.

Corresponding Author:

Ai-lian Chen (lian-ai Chen), 652326303@qq.com , Department of Rehabilitation Medicine, Hunan Provincial People's Hospital (The First Affiliated Hospital of Hunan Normal University), Changsha 410005, Hunan, Peoples R China.

(Note: The name in the article is correct, and the name in parentheses comes from ScholarOne. As ScholarOne cannot modify the name, it is hereby stated in the article)

For peer review only

For peer review only - http://bmjopen.bmj.com/site/about/guidelines.xhtml

Effect of robotic-assisted gait training on gait and motor function in spinal cord injury: a protocol of a systematic review with meta-analysis

Abstract：

Introduction: Robotic-assisted gait training (RAGT) has been reported to be effective in rehabilitating patients with spinal cord injury (SCI). However, studies on RAGT showed different results due to a varied number of samples. Thus, summarising studies based on robotic-related factors is critical for the accurate estimation of the effects of RAGT on SCI. This work aims to search for strong evidence showing that using RAGT is effective in treating SCI and analyse the deficiencies of current studies.

Methods and analysis: The following publication databases were electronically searched in December 2022 without restrictions on publication year: Medline, Cochrane Library, Web of Science, Embase, PubMed, the Cochrane Central Register of Controlled Trials and China National Knowledge Infrastructure. Various combinations of keywords, including ‘motor disorders’, ‘robotics’, ‘robotic-assisted gait training’, ‘Spinal Cord Injuries’, ‘SCI’ and ‘gait analysis’ were used as search terms. All articles on randomised controlled trials (excluding retrospective trials) using RAGT to treat SCI that were published in English and Chinese and met the inclusion criteria were included. Outcomes included motor function, and gait parameters included those assessed by using the instrumented gait assessment, the Berg balance scale, the 10-m walk speed test, the 6-min walk endurance test, the functional ambulation category scale, the Walking index of SCI and the ASIA assessment scale. Research selection, data extraction and quality assessment were conducted independently by two reviewers to ensure that all relevant studies were free from personal bias. In

For peer review only

For peer review only - http://bmjopen.bmj.com/site/about/guidelines.xhtml

addition, the Cochrane Bias Risk Assessment Tool was used to assess the risk of bias. Review Manager V.5.3 software was utilised to produce deviation risk maps and perform paired meta-analyses.

Strengths and limitations of this study

1. This study was the first meta-analysis to systematically evaluate the efficacy and safety of RAGT in the treatment of SCI.

2. The results of this study provided evidence for the treatment of SCI patients and helped therapists and patients to choose appropriate treatment methods.

3. Two reviewers independently conducted research selection, data extraction and quality assessment to ensure that all relevant studies were free from personal bias.

4. The language categories of the research search were only included in English and Chinese, and the final search results would have some bias.

Key words: Spinal Cord Injuries; Motor disorders; Rehabilitation; Robotics, Gait Analysis

Ethics and dissemination: Ethics approval is not required for systematic reviews and network meta-analyses. The results will be submitted to a peer-reviewed journal or presented at a conference.

Trial registration number: PROSPERO (CRD42022319555).

Introduction

Spinal cord injury (SCI) is a serious disabling disease that often causes paraplegia or quadriplegia and affects patient's sensory, motor and autonomic nervous functions.[1 2] SCI leads to various complications, such as pressure ulcers, lung infections and urinary tract infections.[3] It also affects patients' quality of life and living standard and imposes a heavy burden on families and society.[4] It ultimately shortens patients' life expectancy.[5] In addition, the mortality rate of patients with

For peer review only

SCI is higher than that of the general population.[6-8] National statistical data show an increasing incidence rate of SCI annually, and that the incidence rate of SCI per million residents is 9.3 persons/year.[9] During the rehabilitation treatment of SCI, improving the walking ability, self-care ability and self-esteem of patients is an important aspect that helps them return to society and reduces their costs. Therefore, increased exercise capacity of the lower limbs is crucial to daily independence and social reintegration for this population, which mainly functions in standing and walking.[10 11]

Robot-assisted gait training (RAGT) can improve the walking ability,[12] lower limb strength and independence of patients with incomplete SCI.[13] RAGT can also improve balance function and has been gradually applied in patients with SCI.[14] In patients with SCI, robots for lower limb rehabilitation can effectively and safely improve walking ability; reduce pressure ulcers,[15] lung infections,[8] urinary tract infections and other complications;[16] improve dignity; and reduce costs. However, high-quality evidence-based medical studies that systematically evaluated the efficacy of RAGT in the treatment of SCI remain scarce.

Therefore, summarising studies based on RAGT-related factors is critical for the accurate estimation of the effects of RAGT on SCI. This meta-analysis aims to systematically evaluate the efficacy of RAGT in alleviating motor dysfunction and restoring speech ability in patients with SCI based on randomised clinical trials (RCTs), find strong evidence demonstrating that using RAGT is effective in the treatment of SCI and analyse the deficiencies of current studies.

Methods

The protocol of this systematic review was planned and conducted following the Preferred Reporting Items for Systematic Reviews and Meta-Analyses Protocols Guideline and PRISMA

For peer review only - http://bmjopen.bmj.com/site/about/guidelines.xhtml

2020 guidelines and was performed following a protocol registered in PROSPERO (CRD42022319555).[17 18] The plan starts on March 1, 2023 and ends on June 1. The review process is shown in Figure 1.

Search strategy

Two reviewers (Jin-lin Peng and Lei Wang) electronically searched the following publication databases in December 2022 without restrictions on publication year: Medline, Cochrane Library, Web of Science, Embase, PubMed and China National Knowledge Infrastructure. Various combinations of keywords, including 'motor disorders', 'robotics', 'robotic-assisted gait training', 'Spinal Cord Injuries', 'SCI' and 'gait analysis' were used as search terms. The key terms matched the appropriate Medical Subject Heading terms. Presearches were performed. Then, the final search was conducted, relevant journals and references of review articles were manually searched online to identify papers that may have been missed in the electronic database searches.

Eligibility criteria

Inclusion criteria

(1) Study design: Only RCTs were included. (2) Selected population: Participants diagnosed with SCI, namely, individuals with any level of traumatic SCI, regardless of the time since injury, sex and age were included. (3) Type of intervention: The experimental groups received RAGT or RAGT combined with other physical therapies. The control group not received RAGT or received other types of physical therapy. (4) Comparison: The treated subjects were compared at baseline and then with the control or sham-stimulated subjects. (5) Type of outcomes measured: Gait analysis indicators, including gait speed (m/s), step length (cm), double support phase (% walking cycle), single support phase (% walking cycle) and symmetry index; Berg balance scale; ASIA assessment

For peer review only - http://bmjopen.bmj.com/site/about/guidelines.xhtml

scale; Holden walking ability classification (functional ambulation category scale); 10-m walk speed test; 6-min walk endurance test; and WISCI Ⅱ score.

Exclusion criteria

Studies involving animal research, conference research, protocol studies or computer model research and duplicate papers were excluded. Two reviewers (Jin-lin Peng and Lei Wang) independently screened titles and abstracts to identify articles reporting studies that met the inclusion criteria. Then, the full-text versions of the identified articles were obtained and separately screened to ensure that they met the inclusion criteria. Moreover, a third reviewer (Ai-lian Chen) made the final assessment regarding whether or not full-text papers met the inclusion criteria.

Data extraction

A reviewer (Lei Wang) prepared the general information and data collection process by another reviewer (Jin-lin Peng). The format of data collection included research design, participants (number, diagnosis, age and target population numbers in each group), eligibility criteria, intervention used on the research group and control group (i.e. site of stimulation, intensity, number of sessions and time of each session) and outcomes of interest.

Quality assessment

The quality evaluation of the included studies was performed independently by two reviewers (Jin-lin Peng and Lei Wang) and was revised by the third reviewer (Ai-lian Chen). The methodological quality of the intervention studies was assessed using the Physiotherapy Evidence Database (PEDro) scale. The PEDro scale is a valid and reliable measure of the methodological quality of RCTs. This 10-item scale is based on the core criteria for RCT quality assessment.[19] The quality of papers was classified based on the PEDro scale. Studies with scores of less than 6 points were considered

For peer review only - http://bmjopen.bmj.com/site/about/guidelines.xhtml

low-quality studies, whereas those with scores equal to or greater than 6 points were considered high-quality studies (scores of 6–7 indicate good quality and 8–10 indicate excellent quality). [20] The GRADEpro GDT online tool was used to evaluate the level of evidence quality of the outcome indicators. The tool is available at its official website http://www.guidelinedevelopment.org/. The GRADEpro GDT online tool for evaluating the quality of outcome indicators includes five degrading factors, namely, risk of bias, inconsistency, indirectness, imprecision and other considerations.[21] The quality of evidence can be divided into four levels, namely, 'high', 'moderate', 'low' and 'very low'.[22]

Risk-of-bias assessment of individual studies

The quality of the included studies was evaluated and their scores were compared in a consensus meeting between two independent authors (Jin-lin Peng and Lei Wang) to minimise errors and potential biases in the evaluation. However, in the event of any disagreement, a third author (Ai-lian Chen) was included in the discussion for a final consensus. The Cochrane Risk of Bias 2.0 tool was used to assess the articles' risk of bias.[23] Each article was assessed for selection bias (random sequence generation and allocation concealment), performance bias (blinding of participants and personnel), detection bias (blinding of outcome assessment), attrition bias (incomplete outcome data reporting) and reporting bias (selective outcome reporting). Each domain was rated as high risk of bias, unclear of bias or low risk of bias. The risk map of the biases of the studies' quality was prepared with Review Manager 5.3.

Patient and Public Involvement

No patient participated in writing the system review plan. However, the results were disseminated to patients with SCI.

For peer review only - http://bmjopen.bmj.com/site/about/guidelines.xhtml

Statistical analysis

A meta-analysis was conducted using Review Manager 5.3. Heterogeneity between studies was evaluated based on the I^2 statistic for the quantification of the proportion of the total outcome attributable to variability amongst studies. The following ranges were defined: I^2 = 0%–30% (no heterogeneity), I^2 = 30%–49% (moderate heterogeneity), I^2 = 50%–74% (substantial heterogeneity) and I^2 = 75%–100% (considerable heterogeneity).[24] Based on heterogeneity, a random-effects model was used when I^2 > 30%, and a fixed-effects model was utilised when I^2 = 0%–30%.

For the comparison of data from different scales, pooled statistics were calculated using standardised mean differences (SMDs). Furthermore, means and standard deviations after intervention and follow-up evaluation for the RAGT and control groups (when relevant) were applied to compute SMDs.

Addressing missing data

Regarding missing data, the original author was contacted for additional information. In the absence of a reply, the data was calculated based on the availability factor. The potential effect of the missing data on meta-analysis results was tested through sensitivity analysis.

Subgroup analysis

Analysis results showed a situation wherein heterogeneity was high and subgroup analysis was required. Grouping analysis was conducted based on age (children, adolescents, middle-aged and elderly), SCI level (cervical, thoracic and lumbar), disease course (recovery and sequelae), treatment prescription and treatment duration to address potential heterogeneity and inconsistency. A meta-analysis was also conducted to explore possible sources of heterogeneity.

Sensitivity analysis

For peer review only - http://bmjopen.bmj.com/site/about/guidelines.xhtml

Sensitivity analysis was conducted on the main results to assess the effect of method quality, research quality, sample size, missing data and analysis methods on the results of this review to verify the robustness of the research conclusion.[25]

Assessment of publication bias

Each included study was evaluated based on the PEDro scale. Funnel charts were used to assess the publication bias of the main results included in the study. However, when the funnel chart was asymmetrical, attempts were made to explain its asymmetry.[26]

Discussion

RAGT can improve the walking ability of patients with incomplete SCI and can be used by patients with stable vital signs. For patients with complete SCI, RAGT primarily acts to maintain the range of motion of joints. In recent years, there is an increasing number of studies on using RAGT to improve walking ability in SCI, and the new exoskeleton robot for lower limb rehabilitation has shown the advantage of safe transfer. Our current query shows that our work is the first systematic review and meta-analysis on RAGT for patients with SCI. The results of this meta-analysis could help patients and therapists select the appropriate treatment method for SCI and improve new options based on the comparative evidence for effectiveness and safety. Therefore, we hope that the results of this study will provide evidence for guideline recommendations.

Study limitations

Articles published in both Chinese and English were included. Articles in other languages were not included, and their exclusion may affect our research. When incorporating outcome indicators, all data were sourced from scale evaluation and gait analysis instruments. The lack of research results on neural mechanisms may have had a certain effect on this study.

For peer review only - http://bmjopen.bmj.com/site/about/guidelines.xhtml

Data Availability

The datasets used and analysed in the current study are included in this article.

Ethical Approval

This research is a review, does not involve ethical issues and did not apply for ethical approval.

Funding

This study has no funding support.

Disclosure

All authors have read and approved the final manuscript.

Contributors

As the first authors, WL and P-JL have made equal contributions to this work. WL and C-AL for research concept and design. WL and P-JL are responsible for data acquisition. WL and P-JL made the draft, and C-AL did the supervision. All the authors approved the publication of the Protocol.

Conflicts of Interest

All authors declare no potential conflicts of interest with respect to the research, authorship and/or publication of this study.

REFERENCES

1. Eckert MJ, Martin MJ. Trauma: Spinal Cord Injury. *Surg Clin North Am* 2017;97:1031-45.
2. Anjum A, Yazid MD, Fauzi Daud M, *et al.* Spinal Cord Injury: Pathophysiology, Multimolecular Interactions, and Underlying Recovery Mechanisms. *Int J Mol Sci* 2020;21.
3. Stricsek G, Ghobrial G, Wilson J, *et al.* Complications in the Management of Patients with Spine Trauma. *Neurosurg Clin N Am* 2017;28:147-55.
4. Zhang JM, Li N, Zhu L, et al. Effects of pelvic floor biofeedback electrical stimulation combined with lower limb rehabilitation robot training on intestinal function of patients with spinal cord injury. *Journal of Brain and Nervous Diseases* 2021;29:53-7.
5. Xiang XN, Zhong HY, He HC. Research progress of lower limb exoskeleton rehabilitation robot in improving walking ability of patients with spinal cord injury. *Chinese Journal of Rehabilitation Medicine* 2020;35:119-22.
6. Buzzell A, Chamberlain JD, Eriks-Hoogland I, *et al.* All-cause and cause-specific mortality following non-traumatic spinal cord injury: evidence from a population-based cohort study in Switzerland. *Spinal Cord*

For peer review only

For peer review only - http://bmjopen.bmj.com/site/about/guidelines.xhtml

2020;58:157-64.
7. Mirzaeva L, Lobzin S, Tcinzerling N, *et al.* Complications and mortality after acute traumatic spinal cord injury in Saint Petersburg, Russia. *Spinal Cord* 2020;58:970-9.
8. Li R, Ding M, Wang J, *et al.* Effectiveness of robotic-assisted gait training on cardiopulmonary fitness and exercise capacity for incomplete spinal cord injury: A systematic review and meta-analysis of randomized controlled trials. *Clin Rehabil* 2023;37:312-29.
9. Bárbara-Bataller E, Méndez-Suárez JL, Alemán-Sánchez C, *et al.* Change in the profile of traumatic spinal cord injury over 15 years in Spain. *Scand J Trauma Resusc Emerg Med* 2018;26:27.
10. Mahooti F, Raheb G, Alipour F, *et al.* Psychosocial challenges of social reintegration for people with spinal cord injury: a qualitative study. *Spinal Cord* 2020;58:1119-27.
11. Rahimi M, Torkaman G, Ghabaee M, *et al.* Advanced weight-bearing mat exercises combined with functional electrical stimulation to improve the ability of wheelchair-dependent people with spinal cord injury to transfer and attain independence in activities of daily living: a randomized controlled trial. *Spinal Cord* 2020;58:78-85.
12. Grasmücke D, Zieriacks A, Jansen O, *et al.* Against the odds: what to expect in rehabilitation of chronic spinal cord injury with a neurologically controlled Hybrid Assistive Limb exoskeleton. A subgroup analysis of 55 patients according to age and lesion level. *Neurosurg Focus* 2017;42:E15.
13. Holanda LJ, Silva P, Amorim TC, *et al.* Robotic assisted gait as a tool for rehabilitation of individuals with spinal cord injury: a systematic review. *J Neuroeng Rehabil* 2017;14:126.
14. Nam KY, Kim HJ, Kwon BS, *et al.* Robot-assisted gait training (Lokomat) improves walking function and activity in people with spinal cord injury: a systematic review. *J Neuroeng Rehabil* 2017;14:24.
15. Rathore A, Wilcox M, Ramirez DZ, *et al.* Quantifying the human-robot interaction forces between a lower limb exoskeleton and healthy users. *Annu Int Conf IEEE Eng Med Biol Soc* 2016;2016:586-9.
16. Pattanakuhar S, Ahmedy F, Setiono S, *et al.* Impacts of Bladder Managements and Urinary Complications on Quality of Life: Cross-sectional Perspectives of Persons With Spinal Cord Injury Living in Malaysia, Indonesia, and Thailand. *Am J Phys Med Rehabil* 2023;102:214-21.
17. Cumpston M, Li T, Page MJ, *et al.* Updated guidance for trusted systematic reviews: a new edition of the Cochrane Handbook for Systematic Reviews of Interventions. *Cochrane Database Syst Rev* 2019;10:ED000142.
18. Page MJ, McKenzie JE, Bossuyt PM, *et al.* The PRISMA 2020 statement: an updated guideline for reporting systematic reviews. *BMJ* 2021;372:n71.
19. Elbanna ST, Elshennawy S, Ayad MN. Noninvasive Brain Stimulation for Rehabilitation of Pediatric Motor Disorders Following Brain Injury: Systematic Review of Randomized Controlled Trials. *Arch Phys Med Rehabil* 2019;100:1945-63.
20. Maher CG, Sherrington C, Herbert RD, *et al.* Reliability of the PEDro scale for rating quality of randomized controlled trials. *Phys Ther* 2003;83:713-21.
21. Nasser M, Fedorowicz Z. Grading the quality of evidence and strength of recommendations: the GRADE approach to improving dental clinical guidelines. *J Appl Oral Sci* 2011;19:0.
22. Brożek JL, Akl EA, Compalati E, *et al.* Grading quality of evidence and strength of recommendations in clinical practice guidelines part 3 of 3. The GRADE approach to developing recommendations. *Allergy* 2011;66:588-95.
23. Sterne J, Savović J, Page MJ, *et al.* RoB 2: a revised tool for assessing risk of bias in randomised trials. *BMJ* 2019;366:l4898.
24. Higgins JP, Thompson SG. Quantifying heterogeneity in a meta-analysis. *Stat Med* 2002;21:1539-58.

For peer review only - http://bmjopen.bmj.com/site/about/guidelines.xhtml

25. Li J, Zhong D, Ye J*, et al*. Rehabilitation for balance impairment in patients after stroke: a protocol of a systematic review and network meta-analysis. *BMJ Open* 2019;9:e026844.

26. Sterne JA, Sutton AJ, Ioannidis JP*, et al*. Recommendations for examining and interpreting funnel plot asymmetry in meta-analyses of randomised controlled trials. *BMJ* 2011;343:d4002.

Figure: Flow chart of meta-analysis for robotic-assisted gait training in patients with spinal cord injury.

For peer review only

For peer review only - http://bmjopen.bmj.com/site/about/guidelines.xhtml

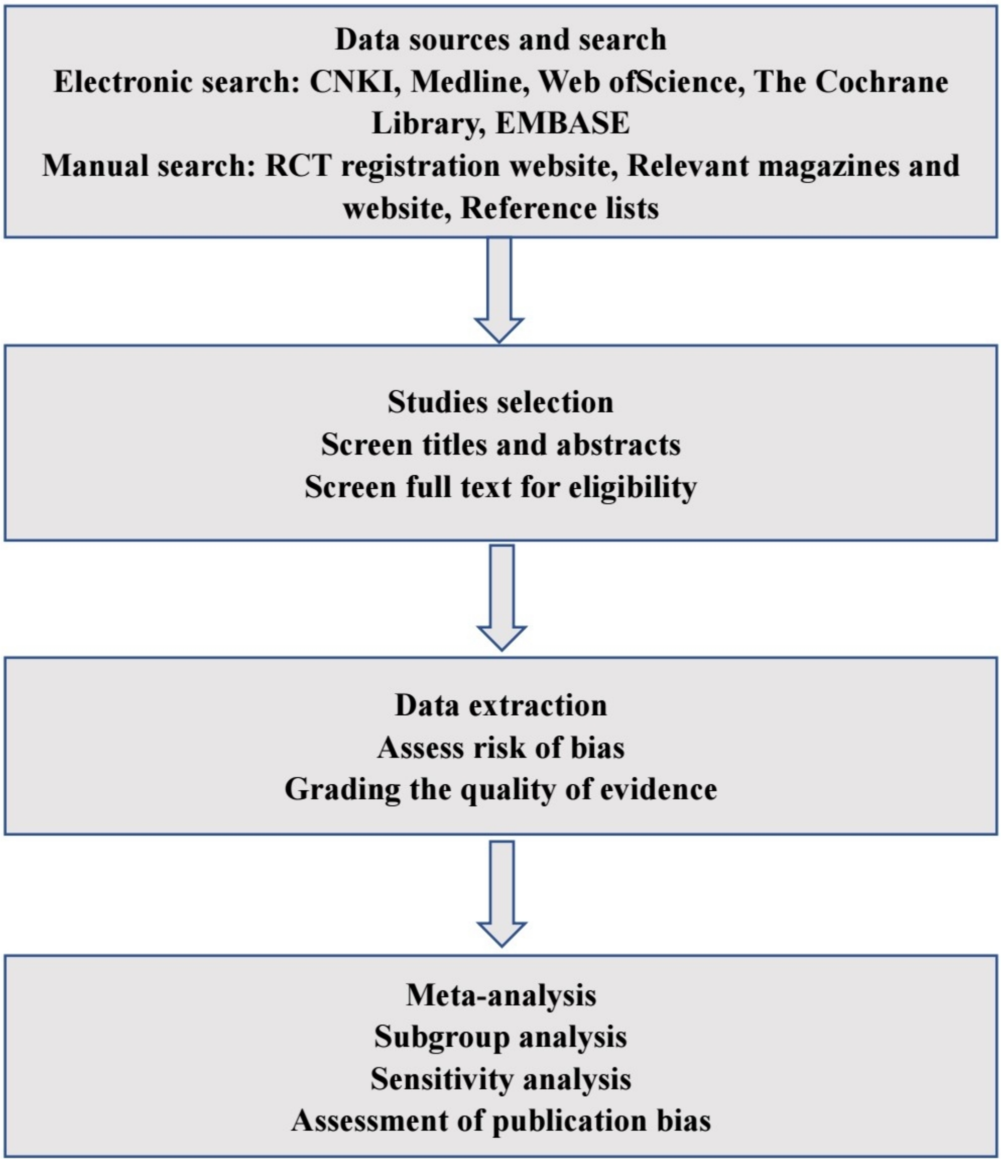

262x303mm (96 x 96 DPI)

PRISMA-P (Preferred Reporting Items for Systematic review and Meta-Analysis Protocols) 2015 checklist: recommended items to address in a systematic review protocol*

Section and topic	Item No	Checklist item	Location where item is reported (line numbers)
**ADMINISTRATIVE INFORMATION**			
Title:			
Identification	1a	Identify the report as a protocol of a systematic review	1
Update	1b	If the protocol is for an update of a previous systematic review, identify as such	
Registration	2	If registered, provide the name of the registry (such as PROSPERO) and registration number	60
Authors:			
Contact	3a	Provide name, institutional affiliation, e-mail address of all protocol authors; provide physical mailing address of corresponding author	4-15
Contributions	3b	Describe contributions of protocol authors and identify the guarantor of the review	212-215
Amendments	4	If the protocol represents an amendment of a previously completed or published protocol, identify as such and list changes; otherwise, state plan for documenting important protocol amendments	
Support:			
Sources	5a	Indicate sources of financial or other support for the review	206-207
Sponsor	5b	Provide name for the review funder and/or sponsor	
Role of sponsor or funder	5c	Describe roles of funder(s), sponsor(s), and/or institution(s), if any, in developing the protocol	
**INTRODUCTION**			**61-85**
Rationale	6	Describe the rationale for the review in the context of what is already known	81-85
Objectives	7	Provide an explicit statement of the question(s) the review will address with reference to participants, interventions, comparators, and outcomes (PICO)	104-113
**METHODS**			
Eligibility criteria	8	Specify the study characteristics (such as PICO, study design, setting, time frame) and report characteristics (such as years considered, language, publication status) to be used as criteria for eligibility for the review	104-113

For peer review only

Information sources	9	Describe all intended information sources (such as electronic databases, contact with study authors, trial registers or other grey literature sources) with planned dates of coverage	92-101
Search strategy	10	Present draft of search strategy to be used for at least one electronic database, including planned limits, such that it could be repeated	92-101
Study records:			
Data management	11a	Describe the mechanism(s) that will be used to manage records and data throughout the review	114-120
Selection process	11b	State the process that will be used for selecting studies (such as two independent reviewers) through each phase of the review (that is, screening, eligibility and inclusion in meta-analysis)	114-120
Data collection process	11c	Describe planned method of extracting data from reports (such as piloting forms, done independently, in duplicate), any processes for obtaining and confirming data from investigators	121-126
Data items	12	List and define all variables for which data will be sought (such as PICO items, funding sources), any pre-planned data assumptions and simplifications	121-126
Outcomes and prioritization	13	List and define all outcomes for which data will be sought, including prioritization of main and additional outcomes, with rationale	121-126
Risk of bias in individual studies	14	Describe anticipated methods for assessing risk of bias of individual studies, including whether this will be done at the outcome or study level, or both; state how this information will be used in data synthesis	142-152
Data synthesis	15a	Describe criteria under which study data will be quantitatively synthesised	121-126
	15b	If data are appropriate for quantitative synthesis, describe planned summary measures, methods of handling data and methods of combining data from studies, including any planned exploration of consistency (such as I^2, Kendall's τ)	156-166
	15c	Describe any proposed additional analyses (such as sensitivity or subgroup analyses, meta-regression)	171-180
	15d	If quantitative synthesis is not appropriate, describe the type of summary planned	167-170
Meta-bias(es)	16	Specify any planned assessment of meta-bias(es) (such as publication bias across studies, selective reporting within studies)	181-184
Confidence in cumulative evidence	17	Describe how the strength of the body of evidence will be assessed (such as GRADE)	181-184

*** It is strongly recommended that this checklist be read in conjunction with the PRISMA-P Explanation and Elaboration (cite when available) for important clarification on the items. Amendments to a review protocol should be tracked and dated.** **The copyright for PRISMA-P (including checklist) is held by the PRISMA-P Group and is distributed under a Creative Commons Attribution Licence 4.0.**

For peer review only

From: Shamseer L, Moher D, Clarke M, Ghersi D, Liberati A, Petticrew M, Shekelle P, Stewart L, PRISMA-P Group. Preferred reporting items for systematic review and meta-analysis protocols (PRISMA-P) 2015: elaboration and explanation. BMJ. 2015 Jan 2;349(jan02 1):g7647.

For peer review only
